# Fluorescent molecularly imprinted polymer particles for glyphosate detection using phase transfer agents

**DOI:** 10.1038/s41598-022-16825-9

**Published:** 2022-08-19

**Authors:** Martha Kimani, Evgeniia Kislenko, Kornelia Gawlitza, Knut Rurack

**Affiliations:** Chemical and Optical Sensing Division (1.9), Bundesanstalt für Materialforschung und -prüfung (BAM), 12200 Berlin, Germany

**Keywords:** Materials science, Polymers, Sensors

## Abstract

In this work, molecular imprinting was combined with direct fluorescence detection of the pesticide Glyphosate (GPS). Firstly, the solubility of highly polar GPS in organic solvents was improved by using lipophilic tetrabutylammonium (TBA^+^) and tetrahexylammonium (THA^+^) counterions. Secondly, to achieve fluorescence detection, a fluorescent crosslinker containing urea-binding motifs was used as a probe for GPS-TBA and GPS-THA salts in chloroform, generating stable complexes through hydrogen bond formation. The GPS/fluorescent dye complexes were imprinted into 2–3 nm fluorescent molecularly imprinted polymer (MIP) shells on the surface of sub-micron silica particles using chloroform as porogen. Thus, the MIP binding behavior could be easily evaluated by fluorescence titrations in suspension to monitor the spectral changes upon addition of the GPS analytes. While MIPs prepared with GPS-TBA and GPS-THA both displayed satisfactory imprinting following titration with the corresponding analytes in chloroform, GPS-THA MIPs displayed better selectivity against competing molecules. Moreover, the THA^+^ counterion was found to be a more powerful phase transfer agent than TBA^+^ in a biphasic assay, enabling the direct fluorescence detection and quantification of GPS in water. A limit of detection of 1.45 µM and a linear range of 5–55 µM were obtained, which match well with WHO guidelines for the acceptable daily intake of GPS in water (5.32 µM).

## Introduction

Glyphosate (GPS) is the most widely used pesticide in the world whose use increased dramatically after the introduction of genetically modified crops engineered to resist its herbicidal action during application^1,2^. In recent years, there have been growing concerns over its toxicity following its classification by the International Agency for Research on Cancer (IARC) as a probable carcinogen^3^ as well as reports of its ecotoxicological effects^4,5^. This has resulted in increased efforts to develop quick and sensitive methods of detection.

Analysis of GPS is complicated due to its high polarity, various prototropic equilibria and the absence of an aromatic group, largely precluding direct spectroscopic measurement. Due to the presence of phosphonic and carboxylic acid as well as amino groups in the molecule, it remains charged over a large pH range^6^. Current established methods for detection and analysis of GPS are based on liquid chromatography (LC) with fluorescence^7,8^ or mass spectrometry detection^9,10^. These approaches usually require derivatization of GPS to impart solubility in the organic solvents used in analysis or to introduce a fluorescent tag on the molecule to facilitate its detection. This is, for example, achieved by reaction with fluorenylmethyloxycarbonyl chloride (Fmoc-Cl), which is a time-consuming step that results in by-products that must be excluded^9^.

Application of molecularly imprinted polymers (MIPs) in sensing applications has grown in importance due to low costs and versatility associated with MIP synthesis and design^11^. Molecular imprinting of GPS has been performed to preconcentrate GPS and its polar metabolites prior to analysis by conventional methods. Puzio^12^ and da Mata^13^ prepared such MIPs for use in column chromatography, while magnetic stir bars have been coated with a MIP layer that could be applied for preconcentration of GPS from aqueous samples^14^. MIP cartridges for solid phase extraction of glyphosate are also commercially available^15^. These approaches, however, still require derivatization of GPS to facilitate its detection.

Detection of GPS using MIPs has been achieved via electrochemical sensing. Mazouz^16^ and Zhang^17^ prepared such MIP sensors for GPS using polypyrrole, while Do^18^ used *p*-aminothiophenol as an imprinting matrix. However, in the latter two examples, other substrates were required to act as reporters for GPS binding, such as hexacyanoferrate/hexacyanoferrite solution as redox probe^17,18^. Although label-free detection of GPS was achieved electrochemically using chitosan-constructed MIPs on gold electrodes, expensive equipment was required for the assay^20^.

Optical detection techniques for GPS employing nanoparticles have also been developed, which also required the use of carefully chosen substrates to enable spectroscopic detection. Sawetwong imprinted GPS on the surface of Mn-ZnS quantum dots, which were then embedded on paper. The binding of GPS inhibited the oxidation of 2,2′-azino-bis(3-ethylbenzothiazoline-6-sulfonic acid) (ABTS) by hydrogen peroxide, resulting in a concentration-dependent color change^19^. Wang coupled carbon nanodots to GPS-binding antibodies and used a competitive assay with antigen-coupled magnetic beads to analyze plant samples^21^. In a dual-nanoparticle system, GPS induced aggregation of silver nanoparticles, resulting in fluorescence recovery of carbon nanodots that were also applied in the system^22^. The same group applied the chelate-forming ability of GPS with Cu^2+^ to realize the turn-on fluorescence of carbon dots incorporated in the assay, allowing for quantification of the herbicide^23^. In related work, GPS induced aggregation in a matrix containing silver nanoparticles and Mg^2+^ by complexation of the ions, resulting in a color change that allowed quantification of the herbicide^24^. More recently, Kim and colleagues combined mesoporous molecularly imprinted silica with embedded quantum dots in which fluorescence quenching was observed upon binding of GPS^25^.

In this work, MIP particles were developed that incorporated a fluorescent probe for direct detection of GPS without further derivatization. This allowed direct quantification of the amount of GPS bound via fluorescence titration experiments and shortened assay times. Moreover, the fluorescence titration data were used to determine binding specificity and binding affinity of the particles to GPS based on the magnitude of the fluorescence changes, simplifying the characterization of MIP binding. Bases consisting of a lipophilic permanent cation (tetraalkylammonium) and a small inorganic anion that drives salt formation (hydroxide, forming water) were used to deprotonate GPS, yielding GPS salts that were soluble in organic solvent where fluorescence sensing occurred. The lipophilic counterions of the bases also served as phase transfer agents, facilitating GPS sensing in mixtures of an organic solvent with water.

Two organic bases were employed to provide counterions for GPS’ acid functions, namely tetrabutylammonium hydroxide (TBA-OH) and tetrahexylammonium hydroxide (THA-OH). To achieve fluorescence detection, a fluorescent crosslinker developed by us^26^ containing urea-binding motifs was used as a probe for GPS-TBA and GPS-THA salts in chloroform, which was a suitable non-polar medium utilized to favor hydrogen bonding between the urea protons of the crosslinker and anionic GPS-TXA moieties. In addition, chloroform is non-miscible with water, thus allowing for analyte extraction prior to indication, a step that commonly improves sensing when sample clean-up is to be avoided as in miniaturized onsite applications. Stable complexes were formed between the GPS analytes and the fluorescent crosslinker, which resulted in bathochromic shifts and enhancements in the signals of the fluorescent reporter in UV/Vis absorption and fluorescence measurements, with association constants in the 10^4^ M^–1^ range. The GPS–fluorescent probe complexes were imprinted into a polymer matrix on the surface of sub-micron silica particles using chloroform as porogen. After a washing step, fluorescent particles imprinted with GPS-TBA and with GPS-THA were obtained, consisting of a 2–3 nm fluorescent polymer shell on the silica core that allowed rapid diffusion of templates/analytes to the binding sites. Due to the incorporation of the fluorescent reporter in the MIP shell, the MIP binding behavior could be easily evaluated by fluorescence titrations in suspension to monitor the spectral changes upon addition of the GPS analytes. We found that the choice of counterion influences the selectivity of the respective MIP particles and is important to facilitate analysis of GPS in water using a biphasic assay, since the phase transfer of the analyte from the aqueous phase to the organic phase requires the molecule to display requisite lipophilicity. With the help of a base such as THA-OH added directly to the aqueous phase to serve as phase transfer agent in such an assay, our approach can be utilized in the detection and quantification of GPS in ground and surface water at concentrations of 5–55 µM. The system can thus be applied in the determination of GPS in water at the concentration set by the WHO as the acceptable daily intake (ADI) (5.32 µM)^27^. The assay can be further optimized to allow miniaturization into microfluidic devices as we previously showed for a comparable system^28^, and therefore shows potential for on-field applications by untrained personnel.

To the best of our knowledge, we report here for the first time MIPs incorporating a fluorescent reporter whose response is directly modulated by (zwitter)ionic GPS and can therefore be used for the direct detection of GPS in organic solvents and in water without the need for prior derivatization of the molecule.

## Materials and methods

### Materials

All organic solvents were purchased from Sigma Aldrich, Acros Organics, abcr, Merck or J.T. Baker and were used without further purification unless otherwise indicated. 2-Isocyanatoethyl methacrylate, analytical grade GPS, analytical grade 3,6-dichloro-2-methoxybenzoic acid (dicamba), 2,4-dichlorophenoxyacetic acid (2,4-D), 3-(methylphosphinico)propionic acid (MPPA), 1 M TBA-OH solution in methanol, 10% THA-OH solution in methanol, tetraethyl orthosilicate (TEOS), 32% ammonia solution in water, (3-aminopropyl)triethoxysilane (APTES), methacrylamide (MAAm), ethylene glycol dimethacrylate (EGDMA), 2,3-diaminophenazine and aluminum oxide type 5016A basic (particle size 50–200 µm) were obtained from Sigma Aldrich/Merck. Triethylamine (TEA) and 37% hydrochloric acid were obtained from AppliChem. 4-Cyano-4-(phenylcarbonothioylthio)pentanoic acid (CPDB) was purchased from abcr. 2,6-Di-tert-butyl-4-methylphenol (BHT) and ethyl chloroformate (ECF) were purchased from Fluka. 2,2′-Azobis(2,4-dimethylvaleronitril) (ABDV) initiator was purchased from Wako Chemicals. Methylphosphonic acid (MPA) was obtained from Alfa Aesar. Milli-Q water was obtained from a Milli-Q ultrapure water purification system (Millipore Synthesis A10).

### Template preparation

GPS, MPA and MPPA were dissolved in 500 µL of Milli-Q water in a 2-mL Eppendorf tube with 10 min of sonication. 2,4-D and dicamba were dissolved in 500 µL of acetonitrile in a 2-mL Eppendorf tube with 10 min of sonication. To this, an equimolar amount of 1 M TBA-OH solution in methanol or 10% THA-OH solution in methanol was added. The mixtures were sonicated for another 10 min, then placed in a vacuum concentrator and dried overnight to give the corresponding templates.

### Synthesis of fluorescent crosslinker I

Fluorescent crosslinker **I**, (((phenazine-2,3-diylbis(azanediyl))bis(carbonyl)) bis(azanediyl))-bis(ethane-2,1-diyl) bis(2-methylacrylate) was synthesized as previously reported with some modifications^26^. Under an argon atmosphere 1 g of 2,3-diaminophenazine (4.3 mmol) and 0.0095 g of BHT (0.4 mmol) were added into a 100 mL round-bottom flask, which was previously dried under vacuum with a heat gun. 35 mL of anhydrous THF was added via a syringe resulting in an orange solution. Thereafter, 1.24 mL of 2-isocyanatoethyl methacrylate (8.6 mmol) was added under stirring under argon and the reaction mixture was heated at 60 °C for 2 h. An additional 1.24 mL of 2-isocyanatoethyl methacrylate (8.6 mmol) was introduced with stirring under argon and the reaction mixture was heated at 60 °C for another 20 h. The reaction was cooled to room temperature and the solvent was evaporated under reduced pressure. Thereafter, the reaction mixture was purified by silica gel column chromatography using dichloromethane/methanol (90:1 → 10:1 v/v) as eluent. A yellow solid was obtained with 11% yield (0.25 g). ^1^H NMR (400 MHz, DMSO-d_6_): δ = 8.48 (s, 2H), 8.40 (s, 2H), 8.13 (ddd, *J* = 12.7, 3.9, 1.3 Hz, 2H), 7.82 (ddd, *J* = 12.8, 3.7, 1.0 Hz, 2H), 7.01 (t, J = 5.7 Hz, 2H), 6.11 (dq, *J* = 1.7, 1.0 Hz, 2H), 5.71 (quintet, *J* = 1.6, 1.6 Hz, 2H), 4.21 (t, *J* = 5.6 Hz, 4H), 3.49 (q, *J* = 5.6, 5.5 Hz, 4H), 1.92 (dd, *J* = 1.5, 1.0 Hz, 6H) ppm. ^13^C NMR (400 MHz, DMSO-D6): δ = 166.6, 155.4, 142.1, 141.1, 136.4, 135.8, 129.5, 128.9, 126.0, 116.7, 63.8, 38.5, 18.0 ppm. UPLCMS-TOF (ESI-) *m/z* calculated for [M-H]^−^ 519.1992, found 519.1913.

### Synthesis of molecularly imprinted polymer particles

#### Synthesis of **MIPTBA@SiO**_**2**_ and **dNIPTBA@SiO**_**2**_

Inhibitor was removed from EGDMA before polymerization by passing the crosslinker through a column packed with basic aluminum oxide. 60 mg of **RAFT@SiO**_**2**_ particles were weighed into 2 separate vials and 3.2 mL of a chloroform solution containing 50.5 µL of EGDMA (83.6 mM), 0.9 mg of fluorescent crosslinker **I** (0.5 mM) and 4.6 mg of MAAm (16.7 mM) were added to each vial. Subsequently, 0.2 mL of 8.9 mM template (GPS-TBA) or “dummy” template (MPA-TBA) solution in chloroform was added to the particle suspension, which was then sonicated for 5 min. 3.6 mM of ABDV initiator solution was prepared in chloroform and 1.8 mL of this was added to each vial. The suspensions were degassed with argon for 15 min and then heated to 50 °C while stirring at 700 rpm. The reaction was left for 24 h, after which the temperature was increased to 70 °C for another 2 h. The particles were precipitated with 4.5 mL of acetonitrile, then washed three times with 5 mL of acetonitrile to remove excess polymer, with centrifugation at 4146×*g* for 5 min and 5 min sonication between washes. To remove templates, the particles were then washed three times with 5 mL of methanol/acetic acid (99:1) for 1 h each on a rotating plate at 40 rotations per minute, with centrifugation at 4146×*g* for 5 min in between. Finally, the **MIPTBA@SiO**_**2**_ and **dNIPTBA@SiO**_**2**_ particles were washed three times with 4.5 mL of acetonitrile with centrifugation at 4146×*g* for 5 min and dried overnight in a vacuum oven. All particles were synthesized twice, and exemplary fluorescence titration measurements are shown in the Supplementary Information to assure the batch-to-batch reproducibility. The procedure yielded the best performing MIPs and dNIPs with a component molar ratio of template/**I**/MAAm/EGDMA = 1:1:30:150. The MIPs and dNIPs synthesized during the optimization process followed the same procedure, with the amounts of **I**, GPS-TBA, MAAm and EGDMA being modified accordingly to achieve different component ratios (see Table S1, Supplementary Information).

#### Synthesis of **MIPTHA@SiO**_**2**_ and **dNIPTHA@SiO**_**2**_

Inhibitor was removed from EGDMA before polymerization by passing the crosslinker through a column packed with basic aluminum oxide. 90 mg of **RAFT@SiO**_**2**_ particles were weighed into 2 separate vials and 4.8 mL of a chloroform solution containing 75.7 µL of EGDMA (83.6 mM), 1.4 mg of fluorescent crosslinker **I** (0.5 mM) and 6.8 mg of MAAm (16.7 mM) were added. 0.3 mL of 7.0 mM template (GPS-THA) or “dummy” template (MPA-THA) solution in chloroform was added to this and the particle suspension was sonicated for 5 min. 3.6 mM of ABDV initiator solution was prepared in chloroform and 2.7 mL of this added to each vial. The suspensions were degassed with argon for 15 min and then heated to 50 °C with stirring at 700 rpm. The reaction was continued for 24 h, after which the temperature was increased to 70 °C for another 2 h. The particles were precipitated with 7.5 mL of acetonitrile, then washed three times with 10 mL of acetonitrile to remove excess polymer, with centrifugation at 12,700×*g* for 5 min and 5 min sonication between washes. To remove templates, the particles were then washed three times with 5 mL methanol/acetic acid (99:1) for 1 h each on a rotating plate at 40 rotations per minute, with centrifugation at 12,700×*g* for 5 min. Finally, the **MIPTHA@SiO**_**2**_ and **dNIPTHA@SiO**_**2**_ particles were washed three times with 10 mL of acetonitrile with centrifugation at 12,700×*g* for 5 min and dried overnight in the vacuum oven. All particles were synthesized twice, and exemplary fluorescence titration measurements are shown in the Supplementary Information to assure the batch-to-batch reproducibility.

### Instrumentation

^1^H and ^13^C NMR spectra were recorded on a Mercury 400 NMR spectrometer (Varian), mass spectra were obtained on an Acquity UPLC (Waters) mass spectrometer with an LCT Premier XE time-of-flight mass detector (Waters), and transmission electron microscope (TEM) images were registered with a Talos™ F200S (200 kV) (Thermo Fisher Scientific). UV/Vis absorption spectra were recorded on a Specord 210 Plus spectrophotometer (Analytik Jena). Steady-state fluorescence measurements were carried out on a FluoroMax-4P spectrofluorometer (Horiba Jobin–Yvon). Standard 10 mm path length quartz cuvettes were used for dye and particle titrations, while circular 100 µm path length cuvettes were used to record prepolymerization spectra using front-face geometry. Zeta potential measurements were performed with a Zetasizer Nano ZS (Malvern Panalytical). Thermogravimetric analyses (TGA) were carried out on a STA7200 (Hitachi High-Tech Analytical Science) thermobalance, using in a first step a nitrogen atmosphere (80 mL min^–1^) with a heating program consisting of a ramp of 10 °C min^–1^ from 25 to 600 °C and in a second step an oxidant atmosphere (air, 80 mL min^–1^) from 600 until 1000 °C with a heating program consisting of a ramp of 10 °C min^–1^. Elemental analysis measurements were performed on a Carbon/Sulfur Analyzer CS-800 (Eltra). BET isotherm data was acquired by N_2_ adsorption/desorption on an ASAP 2010 instrument (Micromeritics).

### Particle characterization

For TEM measurements, a 1 mg mL^–1^ particle suspension was prepared in chloroform, and 9 µL of this placed on a copper grid and left for drying. Images were analyzed with ImageJ software (National Institutes of Health and the Laboratory for Optical and Computational Instrumentation)^29^. To determine the diameter of the particles, data from 100 particles was collected, and the average and standard deviation of the measurements were calculated. To determine MIP and dNIP shell thickness, data from 20 particles was collected, and the average and standard deviation of the measurements were calculated.

For zeta potential determination, a 0.04 mg mL^–1^ particle suspension was prepared in Milli-Q water (pH 6) and the zeta potential was measured using disposable folded capillary cells.

For fluorescence titrations, 1 mg mL^–1^ suspensions of the MIP and dNIP particles were prepared in chloroform; 1 mM solutions of GPS-TBA, MPPA-TBA, 2,4-D-TBA and dicamba-TBA or GPS-THA, MPPA-THA, 2,4-D-THA and dicamba-THA were also prepared in chloroform. Increasing volumes of the 1 mM solutions were added to 2 mL suspensions of the MIP and dNIP particles in a quartz cuvette, and after 2 min of mixing for equilibration, the resultant fluorescence spectra were recorded. For biphasic titrations, 1 mg mL^–1^ suspensions of the MIP and dNIP were prepared in chloroform and 2 mL placed in a quartz cuvette. 1 mL of Milli-Q water was placed on top of the chloroform layer. 1 mM solutions of GPS-TBA, GPS-THA and MPPA-THA were prepared in water. Increasing volumes of the 1 mM solutions were added to the aqueous layer, then the two-phase solution vigorously shaken for transfer of template to the organic phase, and after 10 min to allow the organic phase to settle, the resultant fluorescence spectra were recorded. The relative increase in fluorescence intensity, $\frac{\Delta F}{{F}_{0}}=\frac{ {F}_{x}-{F}_{0}}{{F}_{0}}$, was calculated for each fluorescence spectrum of the MIP and dNIP (where ${F}_{x}$ is the fluorescence intensity at 491 nm for each spectrum after template addition, while ${F}_{0}$ is the fluorescence intensity at 491 nm before addition of template. 491 nm was the wavelength at which the maximum emission intensity was recorded after template addition). The imprinting factors (I.F.) were determined from the corresponding MIP:dNIP ratio of $\frac{\Delta F}{{F}_{0}}$ at the saturation point of the titration. The discrimination factors (D.F.) were determined from ratio of $\frac{\Delta F}{{F}_{0}}$ for GPS-TBA or GPS-THA versus the competing template at the saturation point of the titration.

## Results and discussion

### Interaction of fluorescent crosslinker I with glyphosate

**Figure 1:**
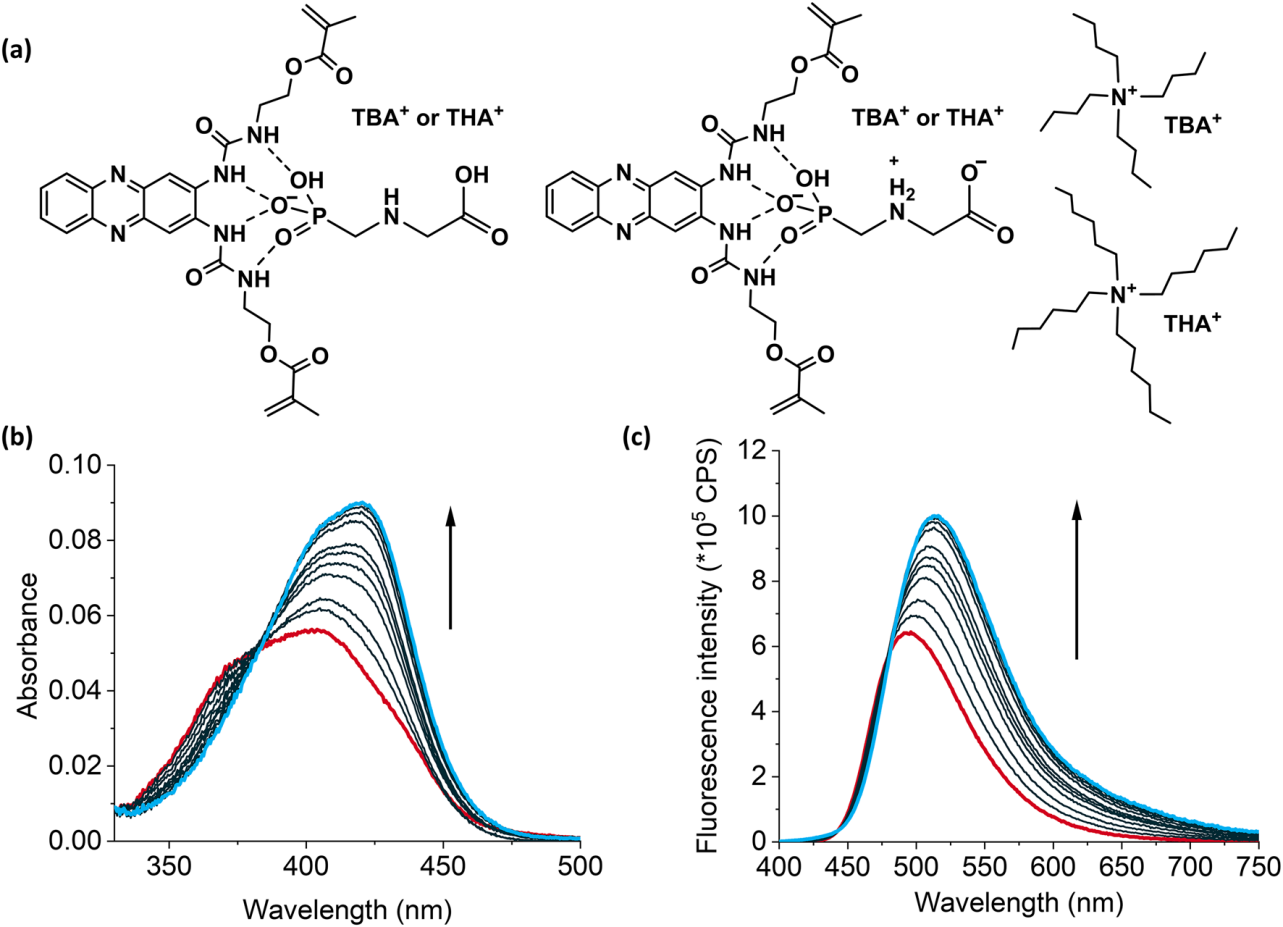
(**a**) Possible modes of interaction between GPS-TXA with **I** in chloroform, showing the structures of TBA^+^ and THA^+^ counterions. (**b**) Absorption and (**c**) fluorescence spectra (λ_ex_ = 385 nm) upon titration of **I** (4.8 µM) with up to 68 equivalents of GPS-TBA in chloroform. Spectra for titration of **I** with GPS-THA are identical and are shown in Fig. S3, Supplementary Information.

Compound **I** is a phenazine-containing fluorescent probe crosslinker which has previously been studied by us (Fig. 1a)^26^. It carries two adjacent urea groups for anion binding and two polymerizable units and possesses fluorescence quantum yields (*Φ*_f_) of 0.02–0.06 in aprotic solvents. Due to the favorable cleft-conformation of the urea groups of the crosslinker, we found it to be a promising tool for the sensing of oxoanions which are located at both termini of GPS. The binding of urea groups to anions occurs by hydrogen bonding and is therefore enhanced in non-polar aprotic solvents. Upon binding of deprotonated phosphates to the urea groups of **I**, increases in absorption and fluorescence signals were observed, along with bathochromic shifts, due to an increase in the electron density in close proximity to the phenazine chromophore, modulating an intramolecular charge transfer process^26^.

Since **I** performed well as a fluorescent probe for oxoanions, we aimed to target the ionic species of GPS for sensing (see Fig. 1a). GPS is a polyprotic molecule carrying different ionizable groups (carboxylate, amino, phosphonate). Although there is still a debate about the different structures and conformations involved in the dye/protonation equilibria as well as the absolute p*K*_a_ data, it has been recently confirmed by various independent methods that the order of successive deprotonation of net neutral GPS is: carboxylate (pK_a1_ = 1.6–2.4), amino (pK_a2_ = 5.2–6.0), phosphonate (pK_a3_ = 9.7–11.0)^30–32^. In neutral water, dissolved GPS can thus form anionic and zwitterionic structures. To facilitate solubility of ionic GPS for interaction with an organic receptor molecule in an organic solvent such as chloroform commonly also used for polymerization, a suitable auxiliary reagent was required. We have previously utilized tetramethylammonium (TMA^+^), tetraethylammonium (TEA^+^), tetrabutylammonium (TBA^+^), tetrahexylammonium (THA^+^) and tetraoctylammonium (TOA^+^) successfully for the imprinting of carboxylates^33^. However, the TMA^+^ and TEA^+^ salts of GPS were still too polar to dissolve in chloroform. Moreover, we previously reported that imprinting with TBA^+^ and THA^+^ salts provided superior selectivity in comparison to TOA^+^, whose alkyl chains were too large for the imprinting of small analytes^33^. We therefore proceeded to utilize GPS-TBA and GPS-THA salts for imprinting and investigated their influence on the interaction mechanism and MIP behavior. To keep the overall species number small, equimolar amounts of TBA-OH or THA-OH were used to ensure that upon redissolution in organic solvents, a monoanionic species TXA^+^/^–^HO_3_P–CH_2_–NH–CH_2_–COOH might result, possibly being accompanied by TXA^+^/^–^HO_3_P–CH_2_–NH_2_^+^–CH_2_–COO^–^ in highly polar organic media. Independent of the structure diversity of GPS, the use of TXA^+^ as counterion enables the possibility to form a stable hydrogen-bonded complex with **I** in organic solvents (Fig. 1a).

Firstly, the binding affinity of **I** to GPS-TBA and GPS-THA was determined via UV/Vis absorption and fluorescence titrations in dilute solution. Although ethyl acetate shows low polarity, GPS-TBA and GPS-THA showed poor solubility in this solvent. Chloroform was therefore chosen for the titrations as it could readily solvate both salts and was successfully applied in previous studies with **I**^26^. Upon the addition of both templates to a dilute solution of **I**, a 19-nm red shift and increase in absorption was observed. Concomitantly, there was a 22-nm red shift along with an increase in fluorescence emission (see Figs. 1b,c and S3, Supplementary Information). For both GPS-TBA and GPS-THA, the interaction fit a 1:1 binding model^34^, with association constants from fluorescence spectra, ${\text{K}}_{\text{a}}^{\text{fluor}}$, determined to be 4.94 (± 0.01) × 10^4^ M^–1^ and 4.76 (± 0.02) × 10^4^ M^–1^, respectively (see Fig. S4, Supplementary Information for further details on binding constant determination). Obviously, even if multiple ionic structures of GPS would be possible in chloroform, dye **I** prefers to bind the phosphonate moiety, containing three hydrogen bond accepting O atoms, due to the cleft-like conformation leading to a 1:1 **I**:GPS-TXA binding. Which of the two forms shown in Fig. 1a, left and right, are present, or if both are present at a certain ratio, is however not expected to massively influence binding of GPS through the phosphonate head group and should thus also not have a negative impact on imprinting. Also, TBA^+^ and THA^+^ should not qualitatively influence the preferential binding of the PO_3_H^–^ moiety. The binding strength of GPS-THA to **I** was slightly lower than that of GPS-TBA. In contrast, previous studies with tetraalkylammonium salts of Z-L-phenylalanine (Z-L-Phe-TMA, Z-L-Phe-TEA, Z-L-Phe-TBA, Z-L-Phe-THA and Z-L-Phe-TOA) in a similar system showed that increasing the size of the counterion led to small increases in the binding strength with the template^33^. Presumably, the fact that a fluorescent probe monomer was used in the earlier mechanistic studies in contrast to the cleft-forming fluorescent probe crosslinker used here accounts for these differences, with the stronger shielding of the interaction site and binding groups in **I**–GPS most likely diminishing the influence of the counterion. In addition, the presence of additional polar groups (a secondary amino and carboxylic acid group) in GPS does not favor any stabilizing hydrophobic interactions between the alkyl chains on the counterion and GPS, further mitigating the counterion influence. To perform stoichiometric imprinting, affinity constants > 10^3^ M^−1^ are required to ensure adequate complexation of the target analyte in the prepolymerization mixture^35^. The binding constants we obtained for **I** with GPS-TBA and GPS-THA were satisfactory in this regard and MIP synthesis could be performed.

### Synthesis and characterization of core–shell fluorescent MIP particles

Compared to conventional bulk MIPs, MIPs synthesized as a thin layer on a substrate facilitate faster diffusion of the analyte due to easier access to the binding sites. Therefore, sub-micron silica particles were chosen as polymer supports for MIP synthesis due to ease of preparation and functionalization. RAFT polymerization was used to enable formation of thin polymer shells on the particle surface. The synthesis of the MIP layer was carried out in chloroform to promote stability of the hydrogen-bonded complex between the analytes and the fluorescent crosslinker **I**.

**Figure 2:**
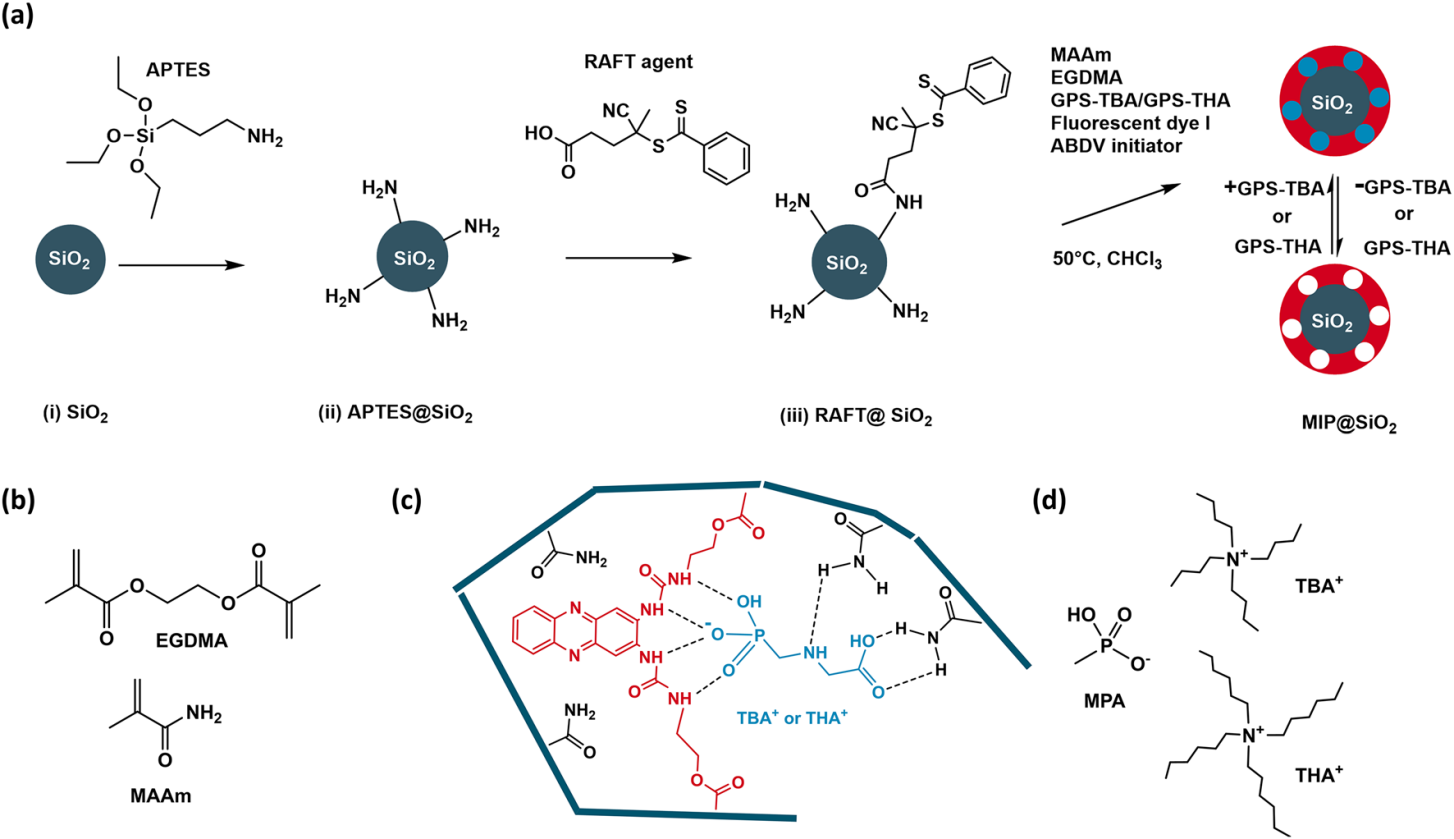
(**a**) Synthesis scheme for MIP particles. (**b**) Comonomer (MAAm) and crosslinker (EGDMA) used in the synthesis of MIP and dNIP particles. (**c**) Incorporation of fluorescent crosslinker, template and comonomer in the polymer matrix. After MIP synthesis, template is extracted to yield binding cavities. Note that hydrogen bonding stabilization by MAAm would happen in a similar way for other possible forms of GPS. (**d**) “Dummy” template (MPA-TBA or MPA-THA) used in synthesis of “dummy” NIPs.

Synthesis and functionalization of the silica particles was done according to our previous work^26^. First, the silica particles were prepared using a modified Stöber method. In the first functionalization step the silica particles were reacted with APTES to form amino groups on the silica particle surface. The terminal amino groups were further modified in a condensation reaction with the carboxylic acid group of the RAFT agent, CPDB (Fig. 2a). The amount of RAFT groups (0.041 mmol g^–1^ of particles) was calculated from the sulfur content determined by elemental analysis. Based on the specific surface area of the silica particles determined from porosimetry (25.95 m^2^ g^–1^), the density of RAFT groups on the particle surface was calculated to be 0.91 molecules nm^-2^ (see Fig. S5, Supplementary Information). The different synthesis and functionalization steps of the silica particles led to changes in the particle surface properties, as revealed by zeta potential measurements (see Fig. S6, Supplementary Information). As expected in purified water at pH 6, silica particles yielded a negative surface charge due to the presence of silanol groups. APTES modification introduced amino groups on the surface leading to a net positive surface charge, whose magnitude was reduced after reaction with CPDB to graft RAFT groups onto the surface.

To create a MIP matrix for binding of GPS templates, MAAm was chosen as functional monomer to participate in hydrogen bonding with the amino and the carboxylate group of GPS-TXA. EGDMA is widely used as a crosslinker in the synthesis of MIPs (Fig. 2b). The combination of **I**, GPS templates, MAAm and EGDMA was expected to lead to the formation of high-affinity cavities for binding of GPS templates in the MIP layer (Fig. 2c). Several MIP recipes were tested to optimize binding performance, and the appropriate component ratio for MIP synthesis that yielded optimal results was determined to be template:**I**:MAAm:EGDMA = 1:1:30:150 (see Table S1, Supplementary Information).

“Dummy” non-imprinted polymer particles (dNIPs) were concurrently synthesized to compensate for non-specific interactions. Conventional NIPs prepared without template possess a random arrangement of functional monomers that could result in high unspecific binding, but the inclusion of a “dummy” template helps to orient the functional groups in a manner that is similar to the MIP, such that the surface characteristics of the resulting polymer shell in the dNIP particles resemble those of the MIPs^36^. Deprotonated methylphosphonic acid (MPA-TBA or MPA-THA) was therefore employed as a “dummy” template for dNIP synthesis (Fig. 2d). The MPA anion, which contains the phosphonic acid group and is a known metabolite of GPS, interacted with **I** in a comparable manner displaying 1:1 stoichiometry^37^. The association constants of MPA-TBA and MPA-THA with **I** were determined by fitting of fluorescence spectra to be 9.52 (± 0.14) × 10^4^ M^–1^ and 4.66 (± 0.02) × 10^5^ M^–1^, respectively (Figs. S7 and S8, Supplementary Information). Contrary to observations made with GPS-TBA and GPS-THA as highlighted above, MPA-THA bound more strongly to **I** than MPA-TBA. MPA is even smaller in size than GPS and contains only a methyl group attached to the phosphonic acid group, which can interact with the alkyl chains on the ammonium ions, so that the binding behavior is presumably influenced to a greater degree by the differences of the counterions.

**Figure 3:**
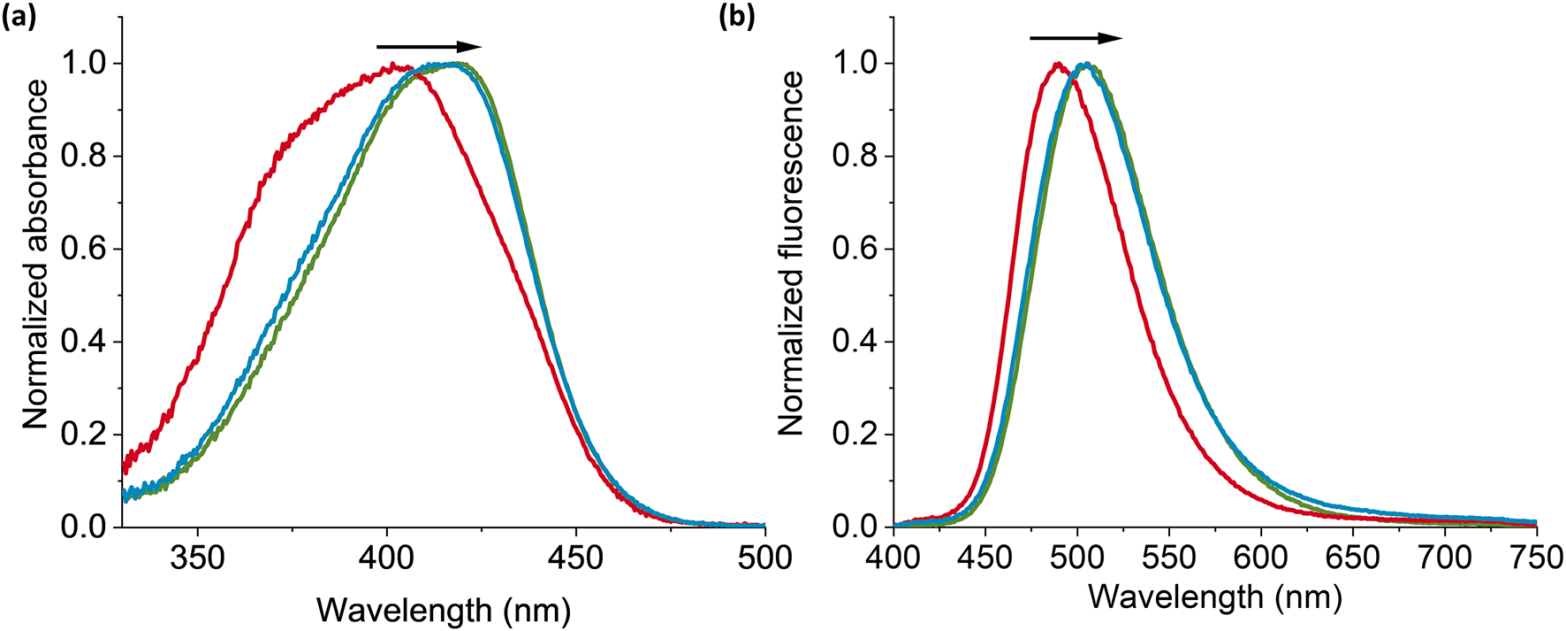
Normalized (**a**) absorption and (**b**) fluorescence spectra of the prepolymerization solutions in chloroform showing spectra of **I** + MAAm + EGDMA (red line), **I** + MAAm + EGDMA + GPS-TBA (blue line) and **I** + MAAm + EGDMA + MPA-TBA (green line). λ_ex_ = 385 nm.

Compared to titrations in dilute solution, higher concentrations of **I** are used in the polymerization reaction (4.8 µM and 343.2 µM, respectively). The stability of the complex of **I** with the GPS and MPA templates in the presence of other polymerization components used in polymer synthesis was crucial to ensure formation of high-affinity binding sites in the MIPs. Consequently, the UV/Vis absorption and fluorescence spectra of the prepolymerization mixtures were recorded in the presence and absence of template to determine if the bathochromic shifts observed in dilute solution after analyte addition were retained at higher concentrations in the presence of MAAm and EGDMA (see Fig. 3 and Table 1, as well as Fig. S9, Supplementary Information). Even in the presence of MAAm and EGDMA, strong bathochromic shifts were observed upon addition of GPS-TBA or MPA-TBA, as was obtained in dilute solution, showing that the hydrogen-bonded complex remained stable under these conditions. The prepolymerization spectra for GPS-THA and MPA-THA with **I** were similar (see Fig. S10, Supplementary Information). MIP particles (with GPS-TBA and GPS-THA) and dNIP particles (with MPA-TBA and MPA-THA) were therefore synthesized, yielding **MIPTBA@SiO**_**2**_, **MIPTHA@SiO**_**2**_, **dNIPTBA@SiO**_**2**_ and **dNIPTHA@SiO**_**2**_, respectively. All synthesized particles were washed with acidic methanol and thereafter with acetonitrile to remove template.

**Table 1:** Bathochromic shifts in absorption (${\lambda}_{\Delta}^{\text{abs}}$) and fluorescence (${\lambda}_{\Delta}^{\text{fluo}}$) maxima (λ_ex_ = 385 nm) after addition of the templates of the prepolymerization mixtures for **MIPTBA@SiO**_**2**_, **MIPTHA@SiO**_**2**_, **dNIPTBA@SiO**_**2**_ and **dNIPTHA@SiO**_**2**_ in chloroform. For comparison**,** the bathochromic shifts in dilute solution in the absence of other polymerization components are included in parentheses.

	${\lambda}_{\Delta}^{\text{abs}}$(nm)	${\lambda}_{\Delta}^{\text{fluo}}$(nm)
**MIPTBA@SiO**_**2**_	12 (19)	11 (22)
**dNIPTBA@SiO**_**2**_	15 (19)	15 (24)
**MIPTHA@SiO**_**2**_	14 (19)	18 (19)
**dNIPTHA@SiO**_**2**_	14 (18)	18 (17)

**Figure 4:**
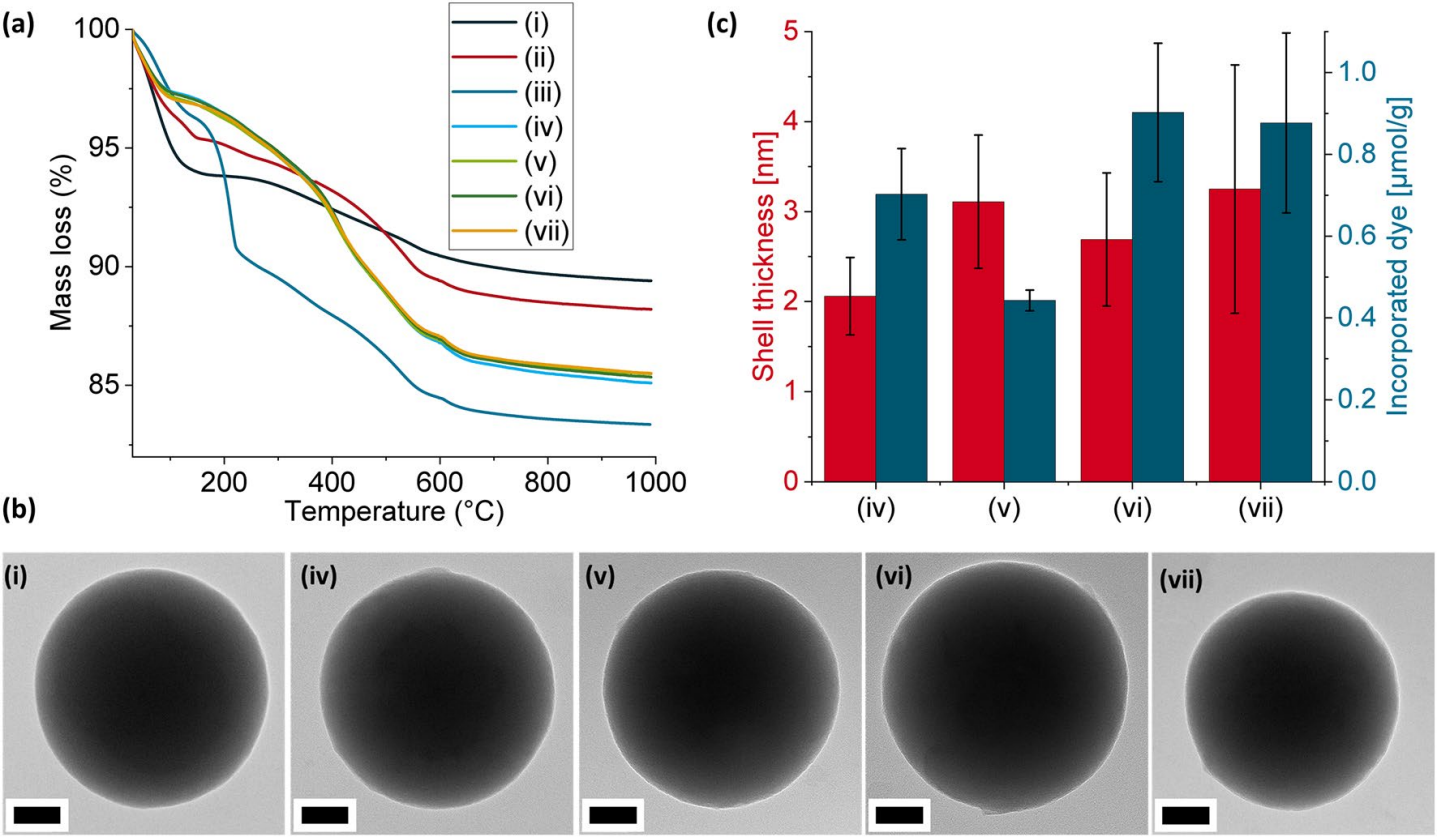
(**a**) TGA curves for (i) SiO_2_, (ii) **APTES@SiO**_**2**_, (iii) **RAFT@SiO**_**2**_, (iv) **MIPTBA@SiO**_**2**_, (v) **dNIPTBA@SiO**_**2**_, (vi) **MIPTHA@SiO**_**2**_ and (vii) **dNIPTHA@SiO**_**2**_. (**b**) TEM images of (i) SiO_2_, (iv) **MIPTBA@SiO**_**2**_, (v) **dNIPTBA@SiO**_**2**_, (vi)** MIPTHA@SiO**_**2**_ and (vii) **dNIPTHA@SiO**_**2**_. Scale bar = 50 nm. (**c**) Shell thickness in nanometers of (iv) **MIPTBA@SiO**_**2**_, (v) **dNIPTBA@SiO**_**2**_, (vi) **MIPTHA@SiO**_**2**_ and (vii) **dNIPTHA@SiO**_**2**_ (red bars), with the amount of incorporated dye (µmol g^–1^) (blue bars).

All particles were characterized by thermogravimetric analysis (TGA) and transmission electron microscopy (TEM). TGA measurements indicated mass loss from combustion of organic groups that were adsorbed or covalently attached on the silica surface. Presumably, the larger mass loss from the **RAFT@SiO**_**2**_ occurring at 150–220 °C compared to the MIP and dNIP particles was due to presence of adsorbed organic groups on the surface of the functionalized particles that were dislodged after MIP synthesis, as we also previously reported (Fig. 4a)^28^.

The diameter of the core silica particles was determined to be 276 ± 19 nm by TEM measurements (Fig. 4b). The thickness of the polymer layer after MIP and dNIP synthesis was determined for each particle type from TEM measurements as well and lay between 2–3 nm, with **MIPTBA@SiO**_**2**_ having the thinnest shells (Figs. 4b, c and S11, Supplementary Information). However, there was no significant difference in shell thickness for the other three particles (**dNIPTBA@SiO**_**2**_, **MIPTHA@SiO**_**2**_ and **dNIPTHA@SiO**_**2**_). To validate that the thin shells allow for faster diffusion of the analyte to the binding sites, the response time of the MIPs to the target template was evaluated by measuring the binding kinetics. Indeed, it was found that the signal increased instantaneously upon addition of the respective template (Fig. S12, Supplementary Information).

The amount of dye incorporated into the polymer network was approximated from the area under the long wavelength band of the absorption spectrum of particle suspensions of known concentration in chloroform, using the respective area of a known concentration of **I** in chloroform as a reference (Figs. 4c, and S13, Supplementary Information). Particles prepared with THA^+^ salts incorporated a larger amount of dye compared with particles prepared with TBA^+^ salts. **MIPTBA@SiO**_**2**_, which had the thinnest shell, contained more dye than **dNIPTBA@SiO**_**2**_. Although the shell thickness of **dNIPTBA@SiO**_**2**_, **MIPTHA@SiO**_**2**_ and **dNIPTHA@SiO**_**2**_ was largely similar, the particles prepared with THA^+^ salts contained nearly two-fold more **I**. THA^+^ possesses longer alkyl chains than TBA^+^ and thus the THA^+^ salts formed a more lipophilic complex with **I** in chloroform which possibly led to an increased incorporation of the dye into the polymer network.

### Binding performance of MIP and dNIP particles in chloroform

The binding capacity of conventional non-fluorescent and other fluorescent MIPs, which, for instance, consist of a fluorescent core such as a quantum dot or carbon dot with a non-fluorescent MIP shell, is determined from incubation of the imprinted particles with template solution followed by quantification of unbound template in the supernatant, which is then used to calculate the amount of bound template. This is a time-consuming process which increases the uncertainty of the measurement from the several steps involved. Due to the incorporation of fluorescent crosslinker **I** into the polymer matrix, the selectivity of the MIP and dNIP particles could be directly evaluated through fluorescence titrations of the particles with the corresponding analytes, since the interaction of **I** with the templates could be easily monitored through the fluorescence increases observed. Fitting of fluorescence data after titration of **I** with GPS-TXA salts in solution in chloroform revealed a 1:1 binding stoichiometry, hence monovalent binding could be expected between the dye-containing binding sites of the MIP particles with the target GPS-TXA salt. For the fluorescence titrations, the particles were suspended in chloroform and the suspension placed in a quartz cuvette. A solution of the template in chloroform was added with increasing concentrations, and the fluorescence changes of the suspension were monitored.

**Figure 5:**
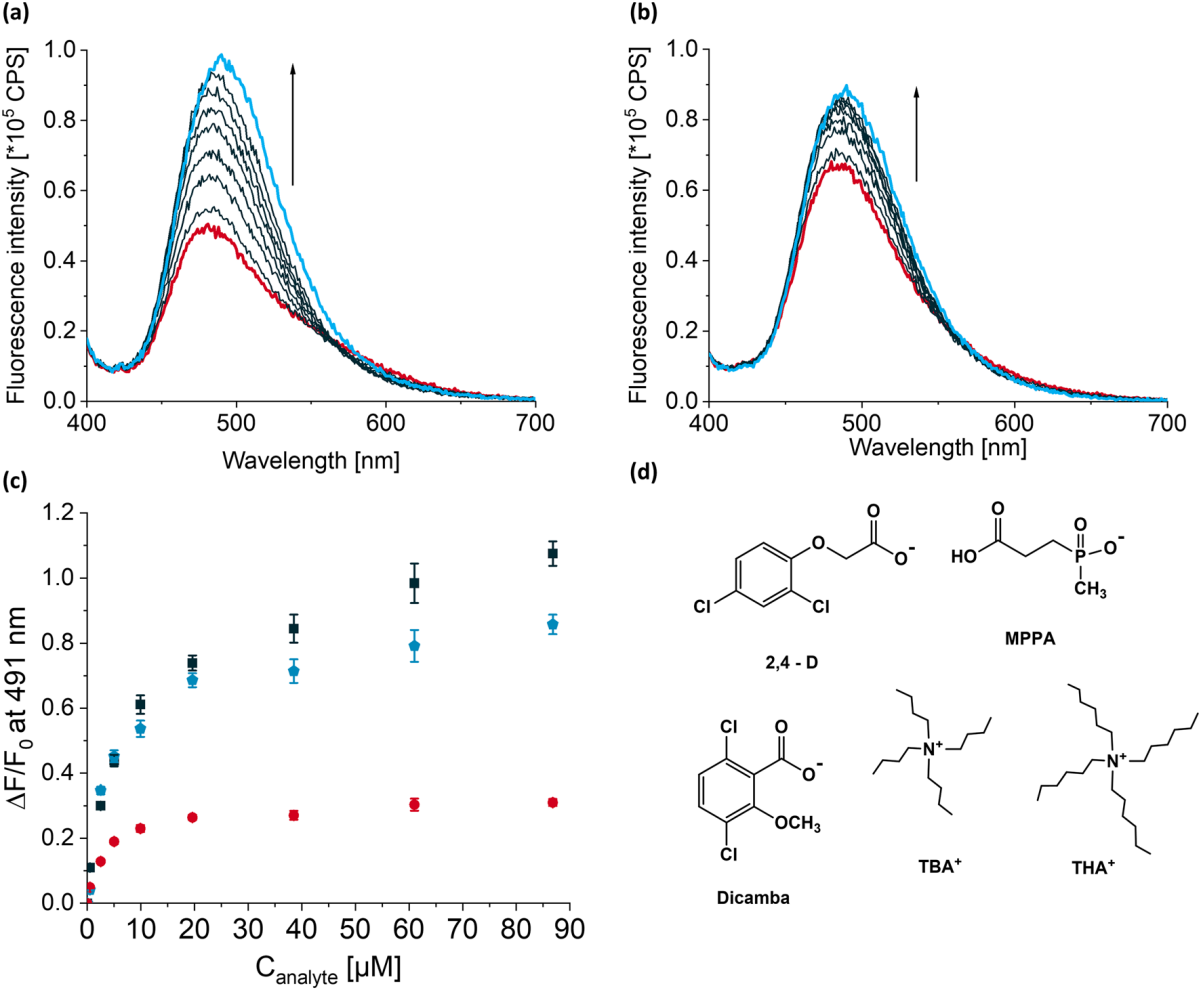
Fluorescence titration spectra of 2 mL each of 1 mg mL^-1^ suspensions of (**a**) **MIPTBA@SiO**_**2**_ and (**b**) **dNIPTBA@SiO**_**2**_ following addition of up to 87 µM of GPS-TBA in chloroform. (**c**) Relative fluorescence changes, $\frac{\Delta \mathrm{F}}{{\mathrm{F}}_{0}},$ at 491 nm after titration of **MIPTBA@SiO**_**2**_ with GPS-TBA (black squares) or MPPA-TBA (blue pentagons) and **dNIPTBA@SiO**_**2**_ with GPS-TBA (red circles). λ_ex_ = 385 nm. (**d**) Chemical structures of MPPA, 2,4-D and dicamba salts used as discriminants in the study.

**MIPTBA@SiO**_**2**_ and **dNIPTBA@SiO**_**2**_ were both titrated against a solution of GPS-TBA. Upon addition of template, hyper- and bathochromic shifts in fluorescence were observed for both **MIPTBA@SiO**_**2**_ and **dNIPTBA@SiO**_**2**_. **MIPTBA@SiO**_**2**_ displayed a stronger fluorescence increase upon addition of GPS-TBA compared to **dNIPTBA@SiO**_**2**_, with an optical imprinting factor (I.F.) of 2.9 being calculated from the respective ratios in fluorescence changes, $\frac{\Delta \mathrm{F}}{{\mathrm{F}}_{0}}$, at the end point of the titration (see Figs. 5a–c, S14, Supplementary Information). Although complete saturation of the fluorescence signal was not achieved for **MIPTBA@SiO**_**2**_, no more than 87 µM of GPS-TBA was added to avoid excessive dampening of the signal due to dilution effects. The association constant of **MIPTBA@SiO**_**2**_ to GPS-TBA was determined by fitting of fluorescence titration data, yielding ${\text{K}}_{\text{a}}^{\text{fluo}}$= 1.49 (± 0.24) × 10^5^ M^–1^, in a 1:1 binding model. The cavities of the MIP displayed stronger affinities for GPS-TBA compared to free **I** in dilute solution, implying successful cavity formation, as we previously showed^36^.

The selectivity of the **MIPTBA@SiO**_**2**_ was evaluated against the TBA salt of MPPA (MPPA-TBA), a known metabolite of GPS. The discrimination factor (D.F.) was calculated from the respective ratios in fluorescence changes at the saturation point of the titration with GPS-TBA and MPPA-TBA, giving a value of 1.33 (see Fig. 5c,d as well as Fig. S15, Supplementary Information). The MIP particles however did not show discrimination against the TBA salts of 2,4-D (2,4-D-TBA) or dicamba (dicamba-TBA) (see Fig. 5d), other commonly used herbicides containing a carboxylate group, which is known to be a potential competitor for urea-type binding motifs (data not shown). In suspension, the fluorometric analytical figures of merit, limit of blank (LOB) and limit of detection (LOD), were calculated to be 0.11 µM and 0.41 µM respectively (see Supplementary Information for details), with a linear dynamic range of 2.5–38.5 µM.

**Figure 6:**
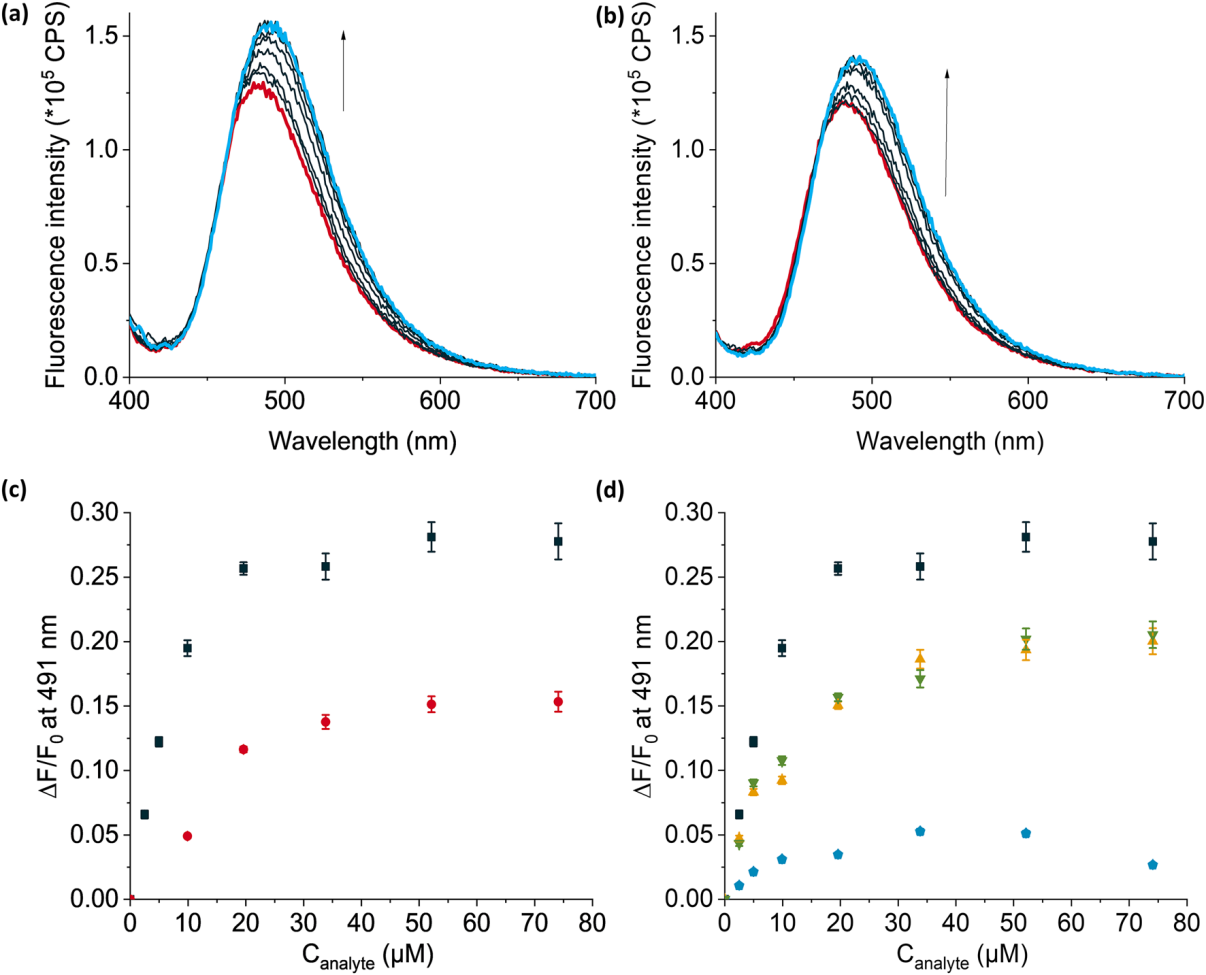
Fluorescence titration spectra of 2 mL each of 1 mg mL^−1^ suspensions of (**a**) **MIPTHA@SiO**_**2**_ and (**b**) **dNIPTHA@SiO**_**2**_ following addition of up to 74 µM of GPS-THA in chloroform. (**c**) Relative fluorescence changes, $\frac{\Delta \mathrm{F}}{{\mathrm{F}}_{0}},$ at 491 nm after titration of **MIPTHA@SiO**_**2**_ (black squares) and **dNIPTHA@SiO**_**2**_ (red circles) with GPS-THA. (**d**) Relative fluorescence changes, $\frac{\Delta \mathrm{F}}{{\mathrm{F}}_{0}},$ after titration of **MIPTHA@SiO**_**2**_ with GPS-THA (black squares), MPPA-THA (blue pentagons), 2,4-D-THA (yellow triangles) and dicamba-THA (green up-side down triangles); for chemical structures of competitors, see Fig. 5. λ_ex_ = 385 nm.

Titration of **MIPTHA@SiO**_**2**_ and **dNIPTHA@SiO**_**2**_ against a solution of GPS-THA resulted in an I.F. of 1.81 (see Figs. 6a–c, S14, Supplementary Information). The association constants of **MIPTHA@SiO**_**2**_ to GPS-THA was determined to be ${\text{K}}_{\text{a}}^{\text{fluo}}$= 8.09 (± 1.59) × 10^4^ M^–1^ from fluorescence spectra, also fitting a 1:1 binding model. As observed for **MIPTBA@SiO**_**2**_, the binding constant of **MIPTHA@SiO**_**2**_ to GPS-THA was enhanced compared to studies in dilute solution, implying successful cavity formation. The D.F. of **MIPTHA@SiO**_**2**_ against MPPA-THA was found to be 5.3, much higher than the D.F. obtained for **MIPTBA@SiO**_**2**_ against MPPA-TBA. Interestingly, **MIPTHA@SiO**_**2**_ could also discriminate against 2,4-D-THA and dicamba-THA, with discrimination factors of ca. 1.4, respectively (see Figs. 6d and S16, Supplementary Information). The LOB and LOD for **MIPTHA@SiO**_**2**_ were calculated to be 1.2 µM and 2.2 µM respectively, with a linear dynamic range of 5.0–52.1 µM. These values were higher than what was obtained for **MIPTBA@SiO**_**2**_, presumably due to the lower affinity constants of **I** to GPS-THA.

### Comparison of particles prepared with TBA and THA counterions

**Figure 7:**
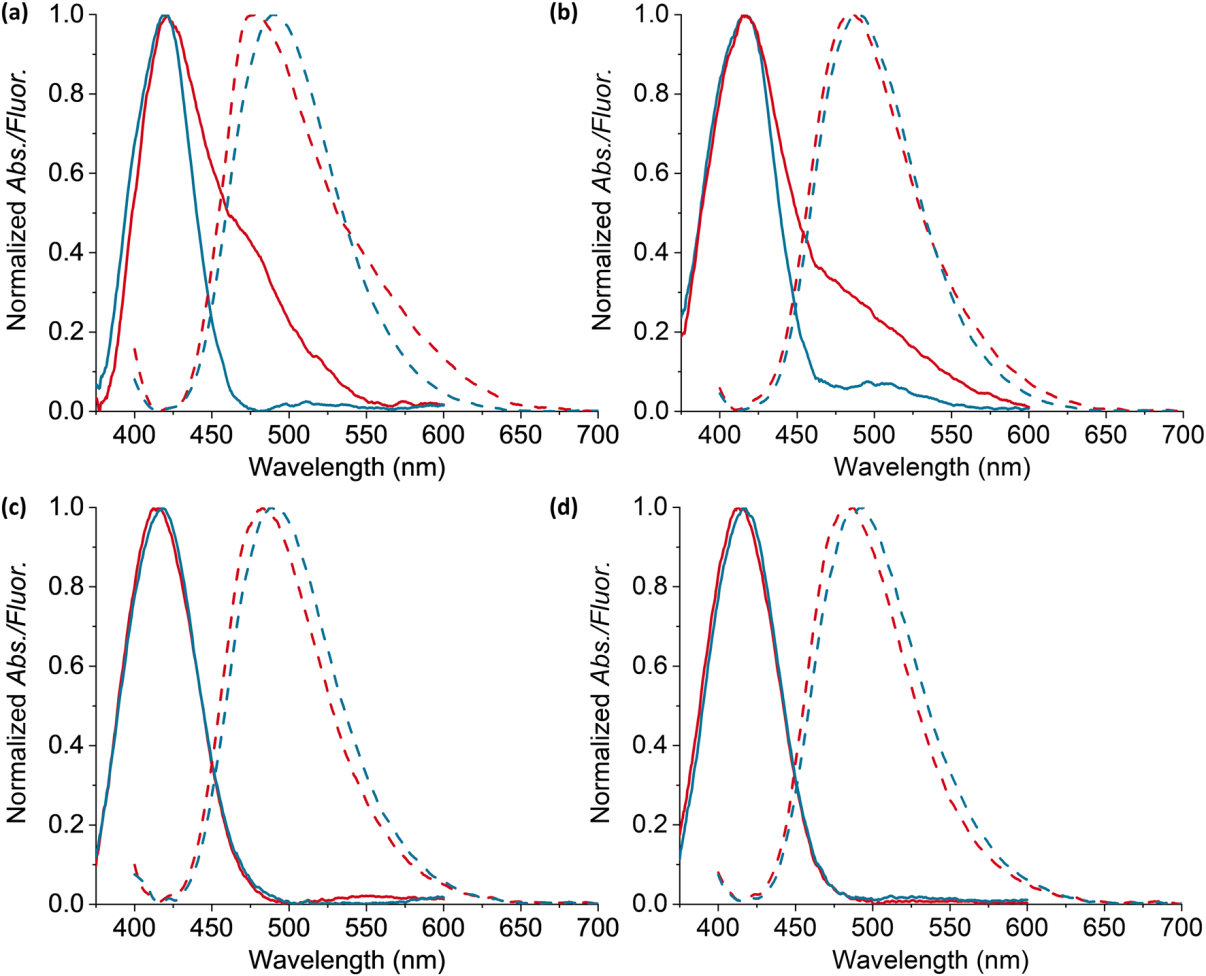
(**a**) Normalized absorption (solid lines) and fluorescence spectra (dotted lines) of **MIPTBA@SiO**_**2**_ before (red line) and after (blue line) addition of 87 µM GPS-TBA in chloroform. (**b**) Normalized absorption (solid lines) and fluorescence spectra (dotted lines) of **dNIPTBA@SiO**_**2**_ before (red line) and after (blue line) addition of 87 µM GPS-TBA in chloroform. The deprotonation bands are centered at 475 nm (absorption) and at 575 nm (fluorescence). (**c**) Normalized absorption (solid lines) and fluorescence spectra (dotted lines) of **MIPTHA@SiO**_**2**_ before (red line) and after (blue line) addition of 74 µM GPS-THA in chloroform. (**d**) Normalized absorption (solid lines) and fluorescence spectra (dotted lines) of **dNIPTHA@SiO**_**2**_ before (red line) and after (blue line) addition of 74 µM GPS-THA in chloroform. The deprotonation bands are absent in absorption and fluorescence spectra of **MIPTHA@SiO**_**2**_ and **dNIPTHA@SiO**_**2**_. Fluorescence spectra were measured with λ_ex_ = 385 nm.

The I.F. of the particles prepared with TBA^+^ salts was approximately 1.5 times higher than that of the particles prepared with THA^+^ as counterion. A closer look at the relative fluorescence changes, $\frac{\Delta F}{{F}_{0}}$, shows that the response is about two-fold greater for **dNIPTBA@SiO**_**2**_ than for **dNIPTHA@SiO**_**2**_ (0.3 and 0.15, respectively) and three-fold greater for **MIPTBA@SiO**_**2**_ than **MIPTHA@SiO**_**2**_ (1.1 and 0.3, respectively). The absorption spectra of washed **MIPTBA@SiO**_**2**_ and **dNIPTBA@SiO**_**2**_ reveal the presence of an additional broad absorption band centered at 475 nm_**.**_ A small shoulder centered at 575 nm is also observed in the corresponding fluorescence spectra (Fig. 7a,b). These features are absent for **MIPTHA@SiO**_**2**_ or **dNIPTHA@SiO**_**2**_ (Fig. 7c,d)_._ We have previously reported that deprotonation of urea-containing dyes occurs in polar environments and coincides with the formation of ca. 100 nm red-shifted bands in the absorption and fluorescence spectra, as well as a reduction in the fluorescence emission intensity of the main band, since the newly formed species is only weakly emissive^26,38^. We therefore attribute these red-shifted bands in the spectra of **MIPTBA@SiO**_**2**_ and **dNIPTBA@SiO**_**2**_ to deprotonation, which suggests a more polar polymer environment for these particles compared to **MIPTHA@SiO**_**2**_ or **dNIPTHA@SiO**_**2**_. We previously reported that when THA^+^ was used in the imprinting of Z-L-Phe, it was preferably incorporated in the surrounding polymer, rather than the counterion contributing to the final morphology of the binding cavity, as was observed with smaller counterions such as TBA^+^^33^. This would lead to the creation of a more lipophilic polymer environment for particles produced with THA^+^, and could thus explain the differences in polarity of the particles produced with TBA^+^ and THA^+^ that we report here.

Upon addition of template to **MIPTBA@SiO**_**2**_ and **dNIPTBA@SiO**_**2**_, hydrogen bonding becomes energetically favored at the expense of deprotonation, allowing the binding to GPS-TBA to occur. As the (weakly emissive) deprotonated species **I**^**−**^ is converted to neutral **I**, the fluorescence intensity of the main band increases, which accounts for the larger relative fluorescence changes during titrations for **MIPTBA@SiO**_**2**_ and **dNIPTBA@SiO**_**2**_ compared to **MIPTHA@SiO**_**2**_ or **dNIPTHA@SiO**_**2**_ (Fig. 5 vs Fig. 6). Indeed, with increasing amounts of template, the shoulder disappears in the spectra of **MIPTBA@SiO**_**2**_ and **dNIPTBA@SiO**_**2**_, further confirming conversion of **I**^**−**^ to **I** (Fig. 7). Comparing the absorption of deprotonation bands for **MIPTBA@SiO**_**2**_ and **dNIPTBA@SiO**_**2**_, stronger deprotonation is observed for the MIP compared to the dNIP (Fig. 7a,b), implying differences in the polarity of the polymer structure for both particles. Addition of GPS-TBA therefore leads to different responses of the two particles: larger decreases in deprotonation (Fig. 7a,b), stronger red shifts and increases in fluorescence intensity (Fig. 5a,b) are observed for **MIPTBA@SiO**_**2**_ compared to **dNIPTBA@SiO**_**2**_, due to the higher extent of deprotonation and selectivity of the MIP for GPS-TBA.

The use of THA^+^ as counterion for MIP synthesis apparently resulted in the formation of better-defined cavities, evident from improved discrimination that was achieved with **MIPTHA@SiO**_**2**_ against relevant discriminants. We previously reported that the use of larger counterions facilitates the formation of tighter binding cavities^33^, which could explain the improved selectivity of **MIPTHA@SiO**_**2**_ to competing analytes compared to **MIPTBA@SiO**_**2**_. In addition, more dye was incorporated into **MIPTHA@SiO**_**2**_ than in **MIPTBA@SiO**_**2**_ (Fig. 4), which could further improve the specificity of interaction with the templates due to the presence of a larger number of specific binding sites. Moreover, the absence of deprotonated species for **MIPTHA@SiO**_**2**_, which is present for **MIPTBA@SiO**_**2**_, could also influence the selectivity of both particles against competing analytes.

### Application of MIP particles to glyphosate analysis in water

GPS is a highly polar molecule which is soluble only in water in its underivatized form^6^. We therefore undertook to apply the synthesized MIP particles for analysis of GPS in water. In previous reports by us, sensory particles synthesized in chloroform were used for analyte detection in aqueous media in biphasic assays^26,28,38^. The particles were suspended in the chloroform phase in a cuvette, while the analyte was added to an aqueous phase above the organic phase. When TBA^+^ or THA^+^ are used as counterions, they served additionally as phase transfer agents. After a mixing step, the analyte partitions into the organic phase where fluorescence sensing occurs. This approach is advantageous, since matrix effects can be minimized by confining interfering hydrophilic ions to the aqueous phase while GPS-TXA salts transition into the organic phase for sensing.

**Figure 8:**
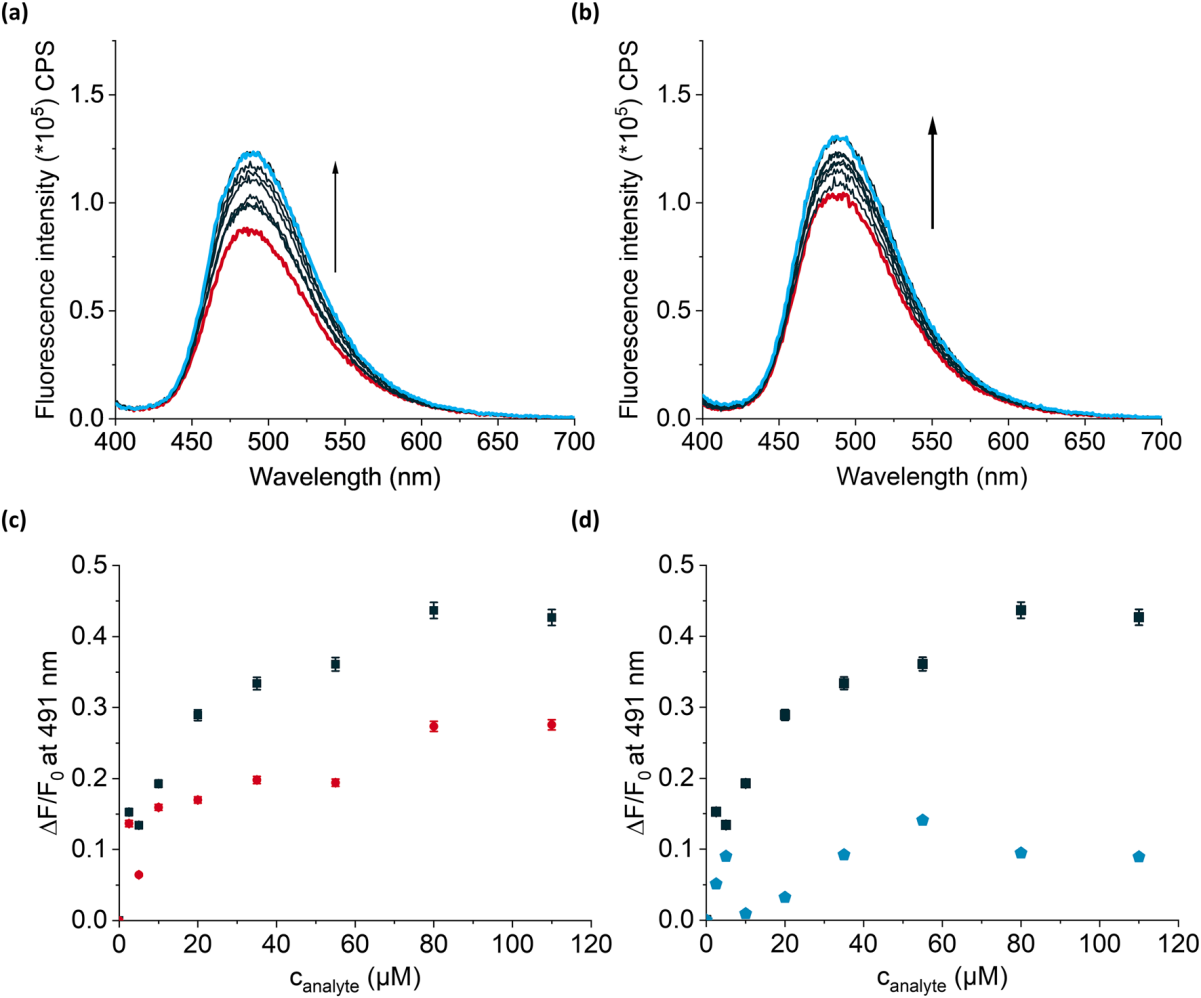
Fluorescence increase of (**a**) **MIPTHA@SiO**_**2**_ and (**b**) **dNIPTHA@SiO**_**2**_ upon titration with up to 110 µM GPS-THA in a biphasic assay. (**c**) Relative fluorescence changes, $\frac{\Delta \mathrm{F}}{{\mathrm{F}}_{0}},$ at 491 nm of **MIPTHA@SiO**_**2**_ (black squares) and **dNIPTHA@SiO**_**2**_ (red circles) upon titration with GPS-THA. (**d**) Relative fluorescence increases of **MIPTHA@SiO**_**2**_ upon titration with GPS-THA (black squares) and MPPA-THA (blue pentagons). 2 mL each of 1 mg mL^-1^ suspensions of particles was used in each experiment, with 1 mL of Milli-Q water added above the chloroform phase. The error bars are given without the contribution from the repetition of the experiments causing the high fluctuations and more detailed information is given in the Supplementary Information. λ_ex_ = 385 nm.

First, we attempted to perform biphasic titrations of **MIPTBA@SiO**_**2**_ and **dNIPTBA@SiO**_**2**_ against GPS-TBA. Interestingly, no fluorescence increases were observed, presumably because GPS-TBA was too hydrophilic to move from the aqueous to the organic phase (see Fig. S18, Supplementary Information). On the other hand, upon titration of **MIPTHA@SiO**_**2**_ and **dNIPTHA@SiO**_**2**_ against GPS-THA, fluorescence increases were recorded that were comparable to what was observed in neat chloroform, showing that GPS-THA possessed the hydrophobicity required to move into the organic phase (Fig. 8). However, the optical I.F. was slightly reduced to 1.6. To confirm that the cross-selectivity of the MIPs was preserved in the biphasic system, **MIPTHA@SiO**_**2**_ was titrated against MPPA-THA (see Fig. S19, Supplementary Information) and the D.F. was found to be 3.0, which was lower than what was obtained in the monophasic system (Fig. 8). The reduction in I.F. and D.F. is to be expected, since the mixing of the two phases introduces traces of water into the organic phase which can form hydrogen bonds to the binding sites of the dye and thus influence the fluorescence response. Moreover, based on its molecular structure, MPPA-THA is less polar than GPS-THA and is expected to have a higher partition coefficient than GPS-THA. The LOB and LOD for the biphasic system were 0.09 µM and 1.45 µM, respectively, which were slightly improved compared to the monophasic system. The linear range of the system for GPS-THA was found to be 5.0–55.0 µM.

To compare the developed system to already developed MIP-based sensors, Table 2 summarizes the performance of several MIP-based sensors for direct detection of GPS in water. Electrochemical detection provides LODs and a linear response in the picomolar and nanomolar range due to high sensitivity of the technique. However, longer run-times (up to 30 min) were required for the assay ^17,18,20^. On the other hand, the colorimetric approach yielded an LOD of 0.018 µM and a linear range of 0.030–296 µM, which is to be expected due to the possible interference from other absorbing species. In total, 7 min were required to run the assay: 2 min for setup of the MIP sensing layer and 5 min for the sensing reaction^19^.

**Table 2:** Examples of MIP-based sensors for direct detection of GPS in water.

MIP synthesis method	Monitoring of GPS binding	LOD (µM)	Linear range (µM)	Assay run-time (min)
Electropolymerized p-aminothiophenol-functionalised gold nanoparticles^18^	Linear sweep voltammetry with a redox probe	4.73 × 10^–9^	5.91 × 10^–9^–5.91 × 10^–3^	20
Electropolymerization of polypyrrole on a gold electrode^17^	Differential pulse voltammetry with a redox probe	1.60 × 10^–3^	2.96 × 10^–2^–4.73 × 10^1^	18
Chitosan MIP electronically deposited on a gold electrode^20^	Electrical impedance spectroscopy	5.91 × 10^–9^	1.83 × 10^–6^–2.96 × 10^–1^	30
Polymer MIP immobilized on Mn/ZnS quantum dots embedded on a paper strip^19^	Oxidation of a substrate by hydrogen peroxide resulting in colour changes	1.18 × 10^–2^	2.96 × 10^–2^–2.96 × 10^2^	7
Fluorescent MIP shell on 270 nm silica core particles (this work)	Fluorescence titrations of MIP particles with GPS-THA in a biphasic system with chloroform/water	9.00 × 10^–2^	5.00 × 10^0^–5.50 × 10^1^	2–3

GPS is a small, highly polar molecule with no sizable absorption in the UV/Vis region and consequently no UV/Vis fluorescence. As a result, previously reported MIP-based techniques for GPS sensing rely on the use of other substrates as reporters for GPS binding, such as a hexacyanoferrate/hexacyanoferrite solution as redox probe^17,18^ or the effect of GPS on the oxidizing potential of hydrogen peroxide^19^. Label-free detection of GPS was achieved electrochemically using chitosan-constructed MIPs on gold electrodes but required expensive equipment. Previously reported methods also required a longer assay run-time (up to 30 min) compared to our approach (only 2–3 min required). To the best of our knowledge, we report here for the first time the direct fluorescence detection of GPS using fluorescent MIPs that respond via a signal change in the presence of GPS and a phase transfer agent. This way, additional reporters for binding events are not required. Instead, the fluorescence emission change upon binding of GPS occurs on the sub-second time scale and can thus be directly recorded in real time, from which a calibration curve can be readily obtained. Our system can thus be applied to determine GPS concentrations of 5.0–55.0 µM in ground and surface water with the help of a base such as THA-OH added directly to the aqueous phase to act as phase transfer agent. This will facilitate transfer of GPS to the organic phase allowing sensing by the MIPs. The acceptable daily intake (ADI) of GPS in water according to WHO guidelines is 0.9 mg L^–1^ (5.3 µM), and our system can therefore be applied for the detection and quantification of GPS within this concentration regime^27^. Moreover, our approach can be further optimized to allow miniaturization into microfluidic devices as we previously reported^28^, and therefore shows potential for on-field applications by untrained personnel.

## Conclusions

We have developed fluorescent MIPs for direct detection of GPS with the aid of phase transfer agents. We present, for the first time, MIPs targeting GPS in which binding of the analyte to the sensing particles results in a measurable fluorescence change due to hydrogen bonding to the urea groups present in the fluorescent probe incorporated into the MIP layer. We show that polymerizable fluorescent reporters are a useful tool for the synthesis of selective MIPs for GPS, particularly allowing for the rapid determination of binding parameters, such as the imprinting and discrimination factors, as well as estimation of binding affinities using spectroscopic data. Moreover, the use of TBA^+^ and THA^+^ as counterions for GPS aids solubility in non-polar solvents, eliminating the need for an extra derivatization step. We found that the choice of counterion is critically important in biphasic assays with water since the analyte should be lipophilic enough to migrate from the aqueous phase to the organic phase for sensing. Our system can be applied to determine GPS concentrations of 5–55 µM in ground and surface water with the help of a base such as THA-OH added directly to the aqueous phase to act as phase transfer agent in a biphasic assay. The dynamic range obtained matches well with WHO guidelines for the ADI of GPS in water. We envision further optimization of the system by adaptation to a droplet microfluidic setup as we previously reported^28^, to speed up kinetics of phase transfer by increasing the surface area of mixing.

## Supplementary Information

Supplementary Information.

## Data Availability

The datasets used and/or analyzed during the current study are available from the corresponding author on reasonable request.
