# Author Correction: Fluorescent molecularly imprinted polymer particles for glyphosate detection using phase transfer agents

**DOI:** 10.1038/s41598-023-28025-0

**Published:** 2023-01-17

**Authors:** Martha Kimani, Evgeniia Kislenko, Kornelia Gawlitza, Knut Rurack

**Affiliations:** Chemical and Optical Sensing Division (1.9), Bundesanstalt für Materialforschung und -prüfung (BAM), 12200 Berlin, Germany

Correction to: *Scientific Reports* https://doi.org/10.1038/s41598-022-16825-9, published online 19 August 2022

The original version of this Article contained an error in Figure 5D, where the chemical structure of MPPA was incorrect. The original Figure 5 and accompanying legend appear below.

**Figure 5:**
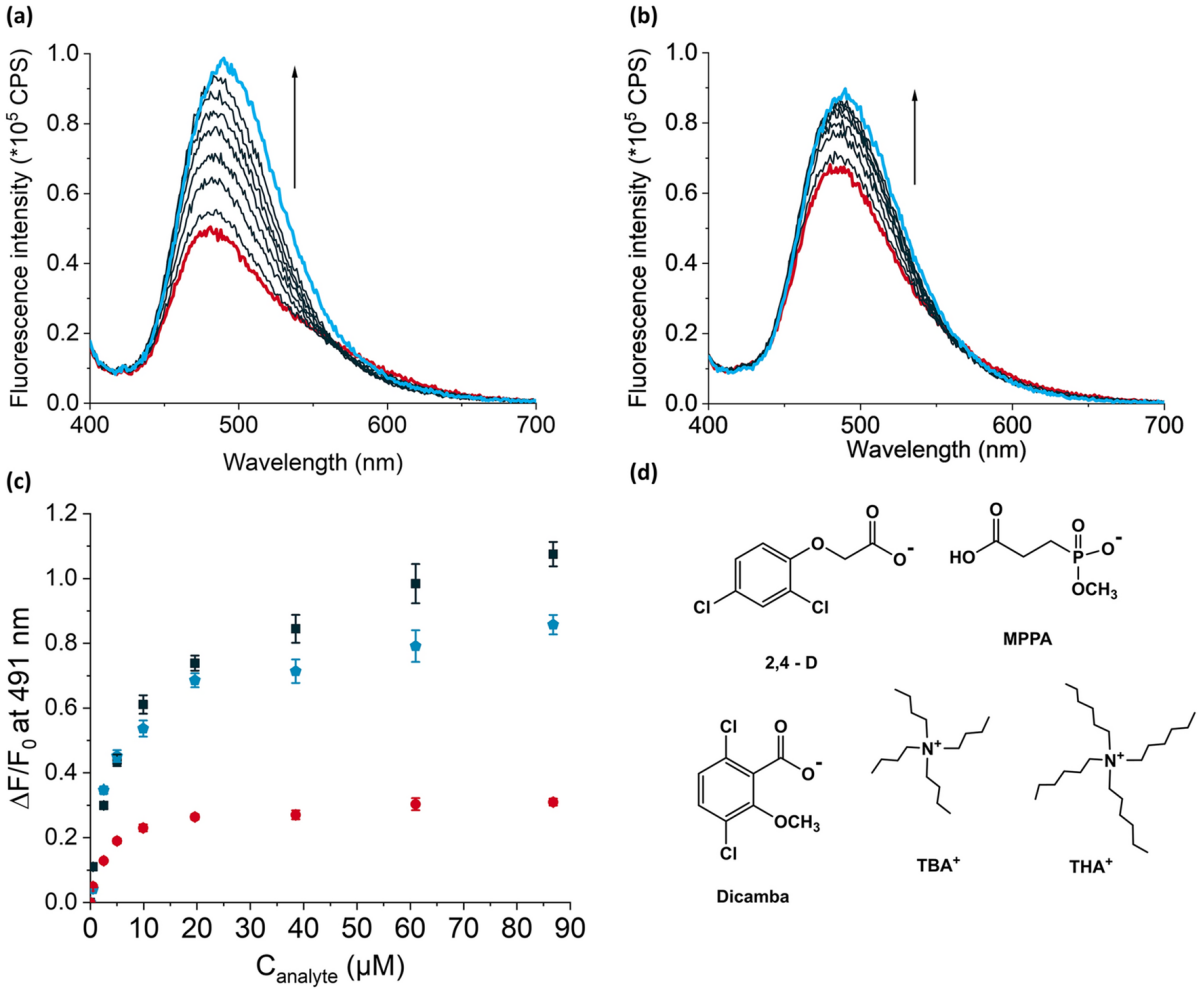
Fluorescence titration spectra of 2 mL each of 1 mg mL^−1^ suspensions of (**a**) **MIPTBA@SiO**_**2**_ and (**b**) **dNIPTBA@SiO**_**2**_ following addition of up to 87 µM of GPS-TBA in chloroform. (**c**) Relative fluorescence changes, $\frac{{\Delta {\text{F}}}}{{{\text{F}}_{0} }}$, at 491 nm after titration of **MIPTBA@SiO**_**2**_ with GPS-TBA (black squares) or MPPA-TBA (blue pentagons) and **dNIPTBA@SiO**_**2**_ with GPS-TBA (red circles). λ_ex_ = 385 nm. (**d**) Chemical structures of MPPA, 2,4-D and dicamba salts used as discriminants in the study.

The original Article has been corrected.

