# Supplementary material for: Fluorescent molecularly imprinted polymer particles for glyphosate detection using phase transfer agents
Source: Sci Rep. 2022 Aug 19;12:14151. doi: 10.1038/s41598-022-16825-9 (PMC9391380; doi:10.1038/s41598-022-16825-9)
Supplement: Supplementary file 1 — Supplementary Information. [file 41598_2022_16825_MOESM1_ESM.pdf]

Electronic Supplementary Information

Fluorescent Molecularly Imprinted Polymer Particles for Glyphosate Detection Using Phase Transfer Agents

Martha Kimani[1], Evgeniia Kislenko[1], Kornelia Gawlitza[1,*], Knut Rurack[1]

[1]Chemical and Optical Sensing Division (1.9), Bundesanstalt für Materialforschung und -prüfung (BAM), Berlin D-12200, Germany

* Correspondence: kornelia.gawlitza@bam.de

Contents:

1 Experimental Section 2
2 NMR spectra of fluorescent crosslinker I 3
3 Absorption and fluorescence spectra of I and GPS-THA 4
4 Determination of binding constant of I with GPS-TBA and GPS-THA 4
5 Determination of surface area of silica core particles by Brunauer-Emmett-Teller (BET) theory 5
6 Zeta potential measurements 6
7 Selectivity of various MIP recipes by varying reactant ratios 6
8 Interaction of I with MPA-TBA and MPA-THA 7
9 Determination of binding constant of I with MPA-TBA and MPA-THA 7
10 Prepolymerization spectra for MIPTBA@SiO_2 and dNIPTBA@SiO_2 8
11 Prepolymerization spectra for MIPTHA@SiO_2 and dNIPTHA@SiO_2 9
12 TEM images of SiO_2 and MIPTBA@SiO_2 10
13 Kinetics of binding for MIPTBA@SiO_2 and MIPTHA@SiO_2 10
14 Determination of amount of I incorporated into MIP and dNIP particles 10
15 Fluorescence titration spectra of reproduced MIPTBA@SiO_2 with GPS-TBA and MIPTHA@SiO_2 with GPS-THA 11
16 Fluorescence titration spectra of MIPTBA@SiO_2 with MPPA-TBA 12
17 Fluorescence titration spectra of MIPTHA@SiO_2 with competitors 12
18 Determination of LOB and LOD of MIPTBA@SiO_2 and MIPTHA@SiO_2 12
19 Fluorescence titration spectra of MIPTBA@SiO_2 with GPS-TBA in the biphasic assay 13
20 Fluorescence titration spectra of MIPTHA@SiO_2 with MPPA-THA in the biphasic assay 14
21 Uncertainty budget calculations 14
22 References 16

1 Experimental Section

1.1 Synthesis of silica beads

Silica particles were synthesized according to the Stöber method with modifications [1]. 65 mL of 96 % ethanol, 121 mL of Milli-Q water and 14 mL of 32 % ammonia solution were mixed at 300 rpm in a 1 L Erlenmeyer flask. 18 mL of TEOS (80 mmol) was mixed with 182 mL of 96 % ethanol and quickly added to the base solution. The mixture was stirred overnight at 300 rpm. The resulting particles were washed 3 times with 96 % ethanol by centrifugation and redispersion at 12,700× *g* for 10 min and then dried overnight in a vacuum.

1.2 Modification of silica particles with APTES

APTES modification of silica particles was performed as previously reported [1]. 1 g of silica particles was weighed into a 2-necked round bottomed flask equipped with a magnetic stirrer and connected to a reflux condenser. The particles were dispersed in 50 mL of anhydrous toluene and heated to 120 °C under argon. 4 mL of APTES (17.1 mmol) was added and the reaction allowed to proceed for 16 h under reflux. The particles were then washed 3 times with 35 mL of 96 % ethanol, with centrifugation at 12,700× *g* for 5 min and 5 min sonication between washes. The particles (**APTES@SiO_2**) were dried overnight in a vacuum.

1.3 Modification of APTES@SiO_2 particles with reversible-addition fragmentation chain transfer (RAFT) agent, CPDB

RAFT modification of the **APTES@SiO_2** particles was performed according to previously published protocols [1]. Briefly, 500 mg of **APTES@SiO_2** particles were weighed into a 20 mL vial equipped with a magnetic stirrer and placed in an ice bath. Simultaneously, 117.4 mg of CPDB (0.4 mmol), 40.3 µL of ECF (0.4 mmol) and 58.7 µL of TEA (0.4 mmol) were dissolved in 8.6 mL of anhydrous tetrahydrofuran (THF) and mixed together in an acetone/liquid nitrogen bath at –78 °C for 40 min. Afterwards, the cooled solution was added to the particles and left to react at room temperature for 24 h at 700 rpm. The particles were precipitated with 15 mL of n-hexane and centrifugation performed at 12,700× *g* for 5 min. The particles were washed once with 20 mL of THF, once with 20 mL of acetone and in 20 mL of THF once more, with centrifugation at 12,700× *g* for 5 min and 5 min sonication between washes. The particles (**RAFT@SiO_2**) were then dried under vacuum overnight.

2 NMR spectra of fluorescent crosslinker I

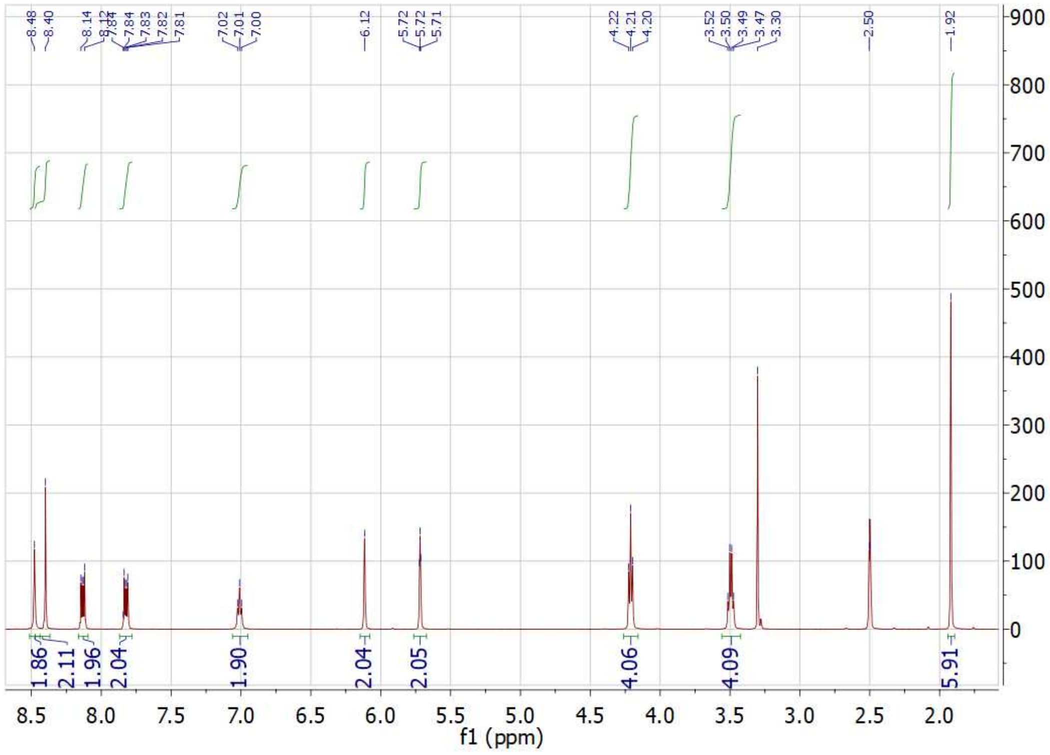

Figure S1. ^{1}H NMR spectrum of **I** in DMSO-d_6.

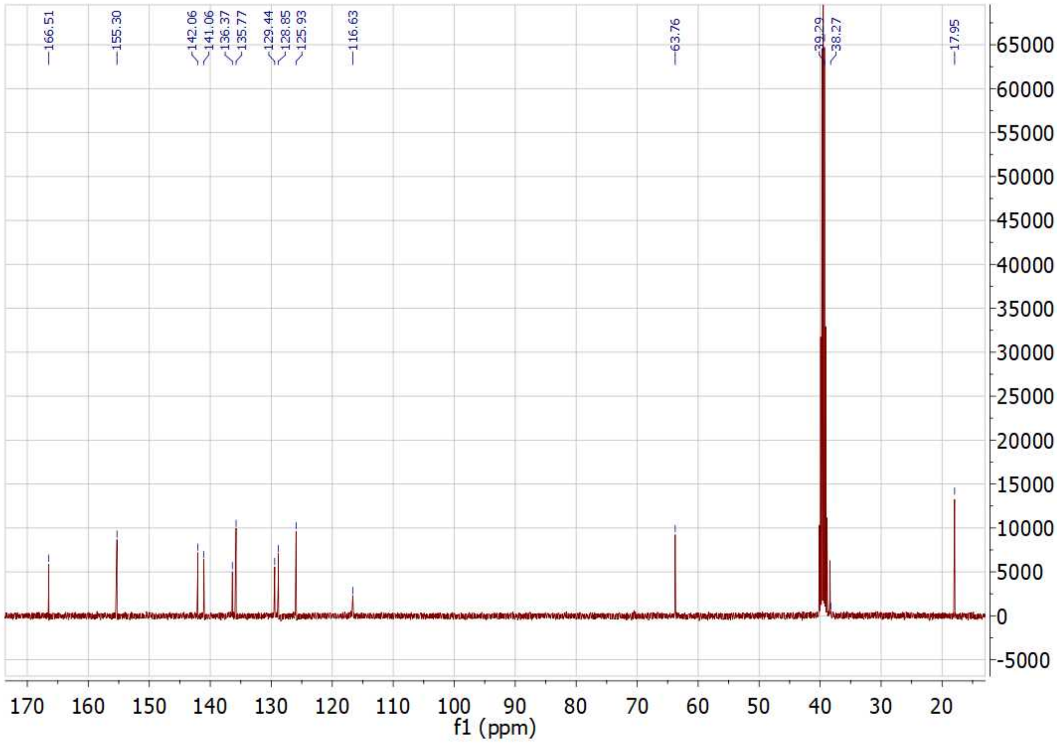

Figure S2. ^{13}C NMR spectrum of **I** in DMSO-d_6.

3 Absorption and fluorescence spectra of I and GPS-THA

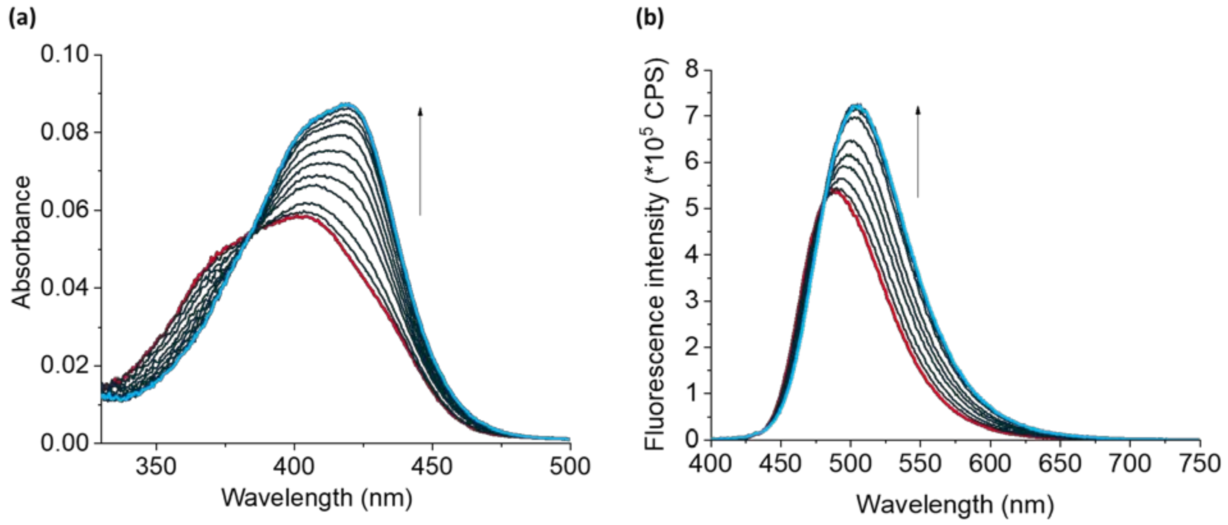

Figure S3. (a) Absorption and (b) fluorescence spectra (λ_{ex} = 381 nm) upon titration of **I** (4.8 µM) with up to 85 equivalents of GPS-THA in chloroform.

4 Determination of binding constant of I with GPS-TBA and GPS-THA

Binding constants were determined using BindFit software (http://supramolecular.org/) [2]. The program accepts input data consisting of host (dye) and guest (GPS-TXA) concentrations as well as fluorescence intensity values at the measured wavelengths. The user provides an initial guess of the binding constants, and the software uses this value to fit the data into a model using non-linear regression. The uncertainty of the fit gives an indication of the suitability of the model obtained, and the initial guess can be refined to obtain a fit with the least uncertainty.

To determine the association constant K_a^{fluor} of **I** with GPS-TBA and GPS-THA, fluorescence spectra were recorded for a dilute solution of **I** in chloroform (4.8 µM), and a solution of GPS-TBA or GPS-THA in this dilute dye solution was prepared (9.6 mM and 3.8 mM, respectively). Small amounts of the template-dye solution were increasingly added to the dilute solution of **I** until saturation was reached. The fluorescence data in both cases fit a 1:1 (host:guest) binding model. The species distribution following the fitting of titration data of **I** with GPS-TBA and GPS-THA is shown in Figure S4.

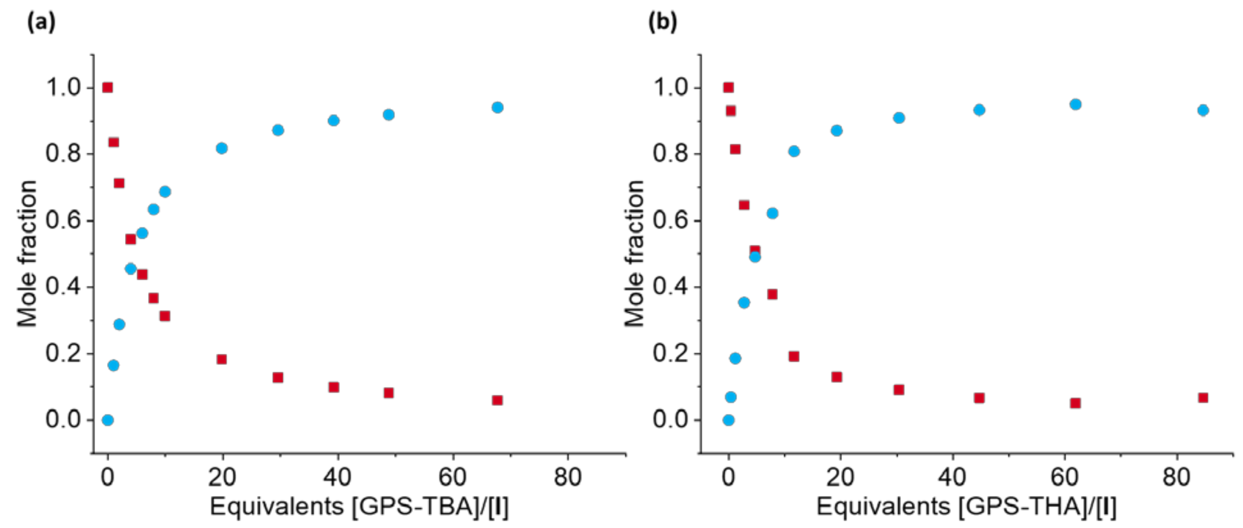

Figure S4. (a) Species distribution of **I** (4.8 µM in chloroform, red squares) and **I**-GPS-TBA complex (blue circles) based on fitting of fluorescence spectra with BindFit®. (b) Species distribution of **I** (4.8 µM in chloroform, red squares) and **I**-GPS-THA complex (blue circles) based on fitting of fluorescence spectra with BindFit®.

5 Determination of surface area of silica core particles by Brunauer-Emmett-Teller (BET) theory

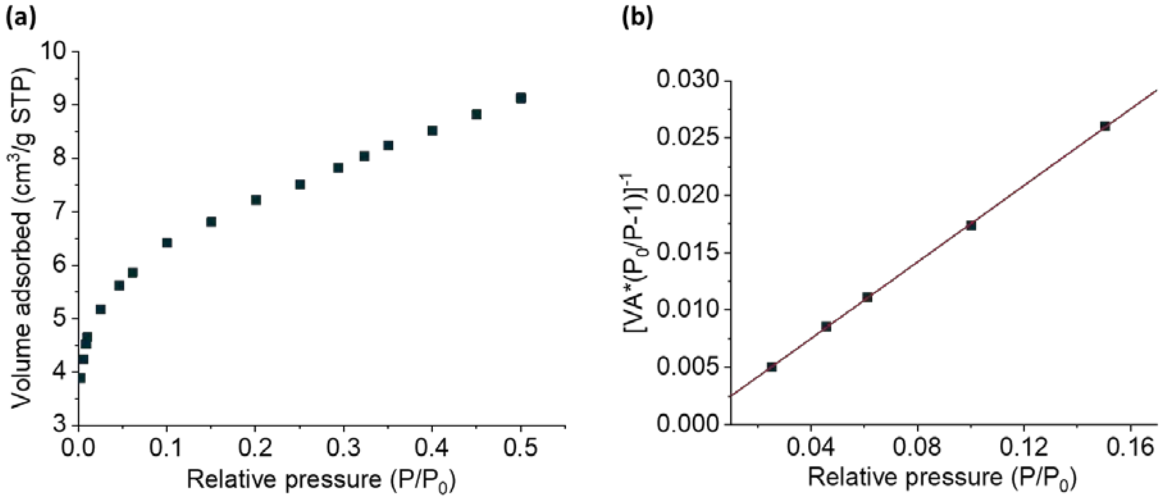

Figure S5. (a) Volume of nitrogen adsorbed per gram of SiO_2 and (b) BET surface area plot.

6 Zeta potential measurements

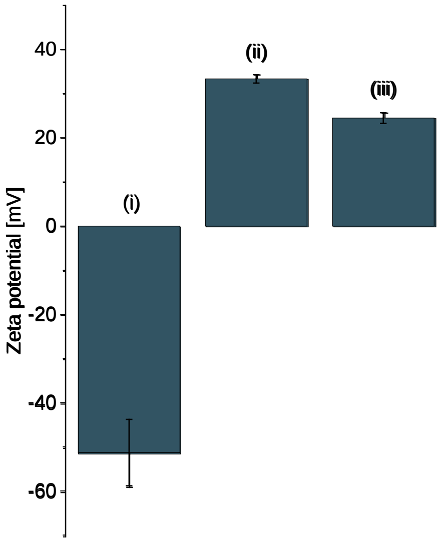

Figure S6. Zeta potential measured at pH 6 of (i) SiO_2 particles, (ii) **APTES@SiO_2**, (iii) **RAFT@SiO_2**.

7 Selectivity of various MIP recipes by varying reactant ratios

Various recipes were tested to optimize binding performance (Table S1). GPS-TBA and MPA-TBA were used as templates to prepare the MIP and dNIP particles respectively. After titration of the particles with GPS-TBA, the relative fluorescence change, $\frac{\Delta F}{F_0} = \frac{F_x - F_0}{F_0}$ was calculated for each fluorescence spectrum of the MIP and dNIP (where F_x is the fluorescence intensity at 491 nm for each spectrum after template addition, while F_0 is the fluorescence intensity at 491 nm before addition of template). The optical imprinting factor (I.F.) was then calculated for each MIP and dNIP combination, by determining the ratio of fluorescence changes of MIP and dNIP at the point of saturation. The molar ratio of template/**I**/MAAm/EGDMA that yielded the best results was 1:1:30:150.

Table S1. Imprinting factors (I.F.) for various MIP recipes using GPS-TBA as template.

**Molar ratio, GPS-TBA:I:MAAm:EGDMA**	**Imprinting factor (I.F.)**
1:1:0:159	1.44
1:1:20:150	1.57
1:1:25:150	1.21
**1:1:30:150**	**2.86**
1:1:35:150	1.31
1:1:40:150	1.45

8 Interaction of I with MPA-TBA and MPA-THA

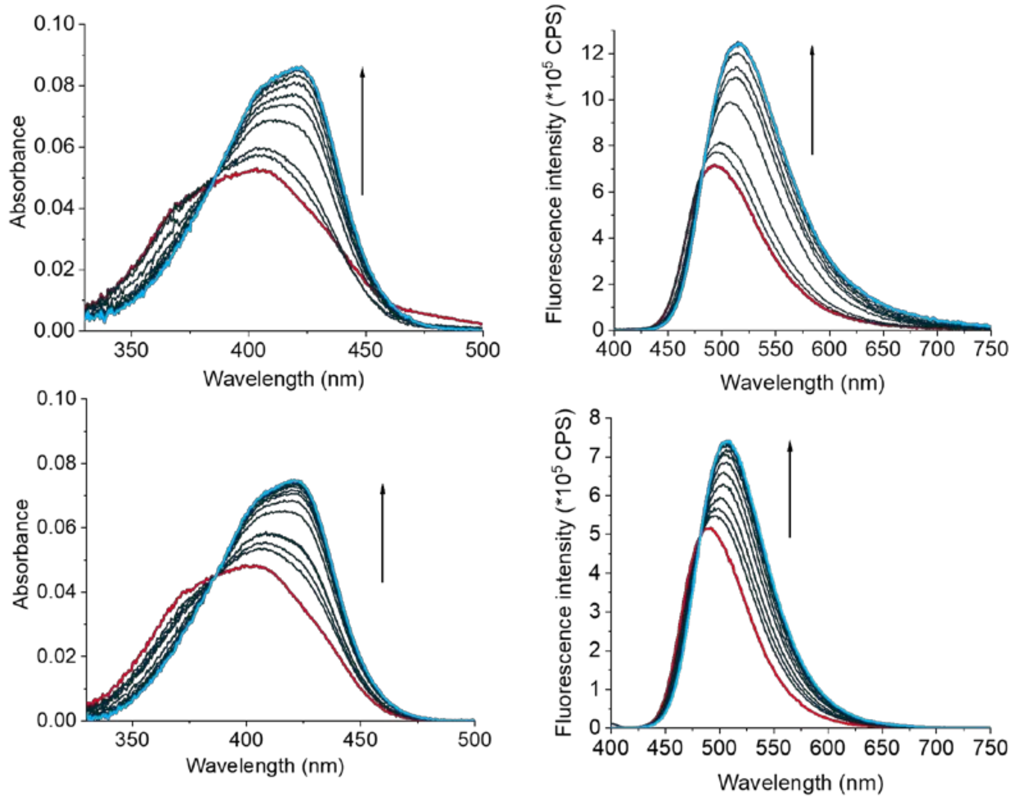

Figure S7. Top: Absorption (left) and fluorescence (right) spectra after titration of **I** (4.8 µM) with up to 15 equivalents of MPA-TBA in chloroform. Bottom: Absorption (left) and fluorescence (right) spectra after titration of **I** (4.8 µM) with up to 4 equivalents of MPA-THA in chloroform. Fluorescence spectra were recorded at λ_{ex} = 385 nm.

9 Determination of binding constant of I with MPA-TBA and MPA-THA

To determine the association constant K_a^{fluor} of **I** with MPA-TBA and MPA-THA, fluorescence spectra were recorded for a dilute solution of **I** in chloroform (4.8 µM), and a solution of MPA-TBA or MPA-THA in this dilute dye solution was prepared (11.4 mM and 6.8 mM, respectively). Small amounts of the template-dye solution were increasingly added to the dilute solution of **I** until saturation was reached. The fluorescence data in both cases fit a 1:1 (host:guest) non-linear binding model using BindFit software [2].

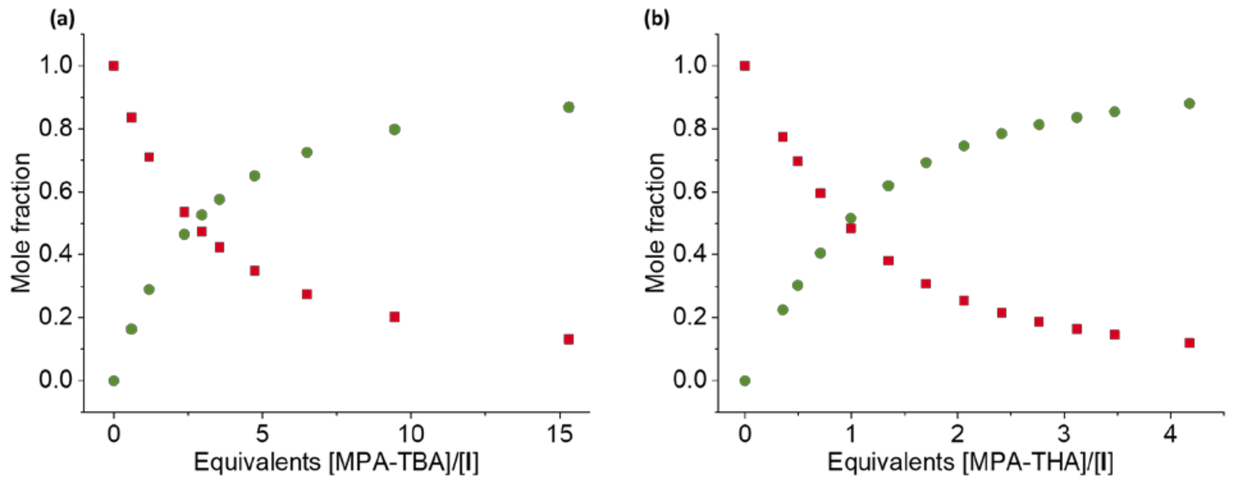

Figure S8. (a) Species distribution of **I** (4.8 µM in chloroform, red squares) and **I**-MPA-TBA complex (green circles) based on fitting of fluorescence spectra with BindFit®. (b) Species distribution of **I** (4.8 µM in chloroform, red squares) and **I**-MPA-THA complex (green circles) based on fitting of fluorescence spectra with BindFit®.

10 Prepolymerization spectra for MIPTBA@SiO$_2$ and dNIPTBA@SiO$_2$

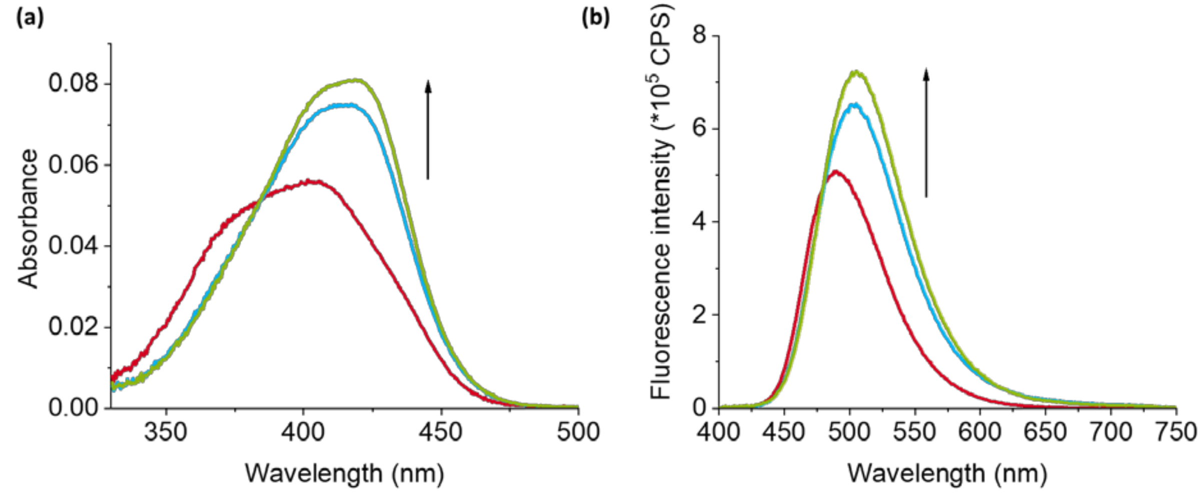

Figure S9. (a) Absorption and (b) fluorescence spectra of the prepolymerization solutions in chloroform showing spectra of **I** + MAAm + EGDMA (red line), **I** + MAAm + EGDMA + GPS-TBA (blue line) and **I** + MAAm + EGDMA + MPA-TBA (green line). λ_{ex} = 385 nm.

11 Prepolymerization spectra for MIPTHA@SiO₂ and dNIPTHA@SiO₂

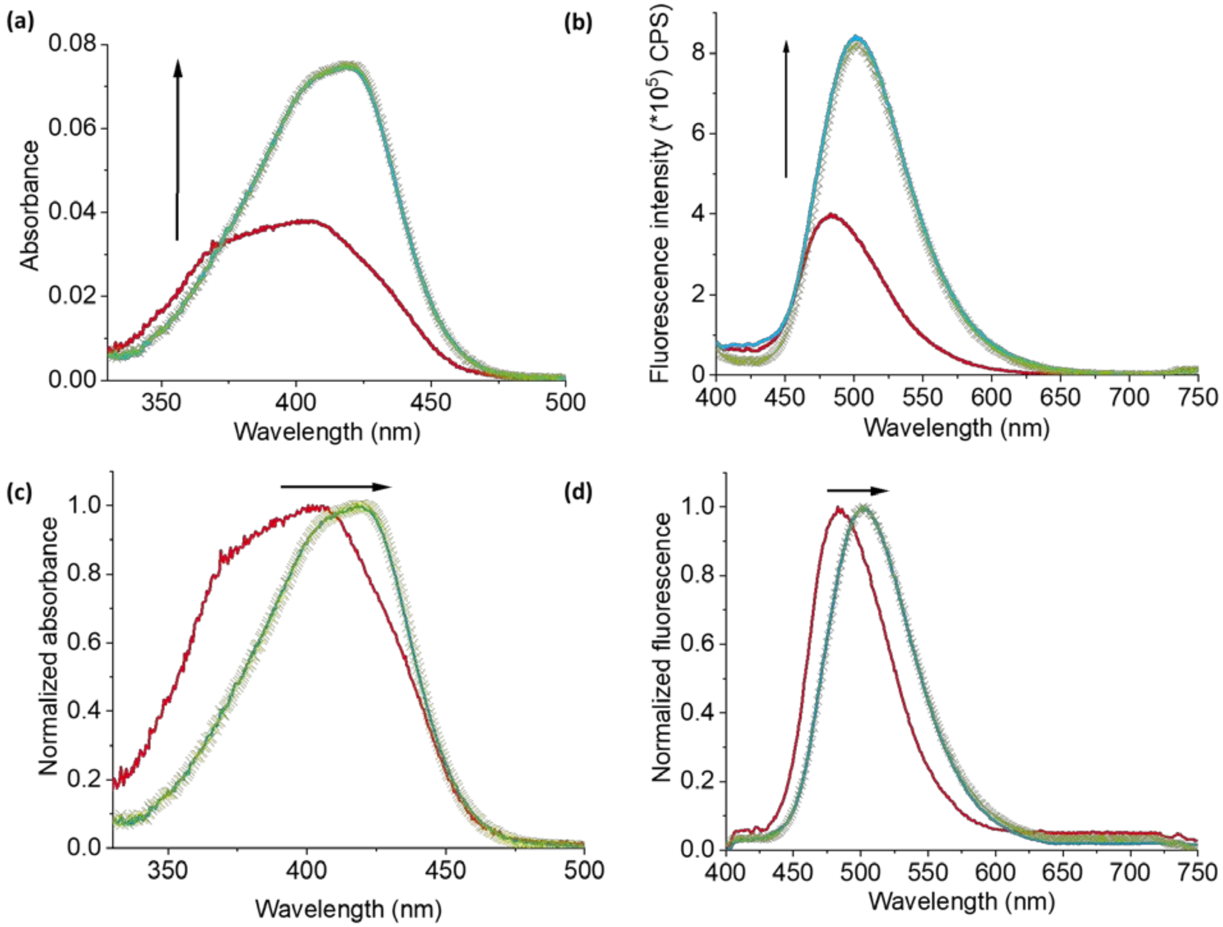

Figure S10. (a) Absorption and (b) fluorescence spectra of the prepolymerization solutions in chloroform showing spectra of **I** + MAAm + EGDMA (red line), **I** + MAAm + EGDMA + GPS-THA (blue line) and **I** + MAAm + EGDMA + MPA-THA (green crosses). The spectra for the complexed species (**I**-GPS-THA and **I**-MPA-THA) overlap largely. The corresponding normalized (c) absorption and (d) fluorescence spectra are also shown. λ_{ex} = 385 nm.

12 TEM images of SiO_2 and MIPTBA@SiO_2

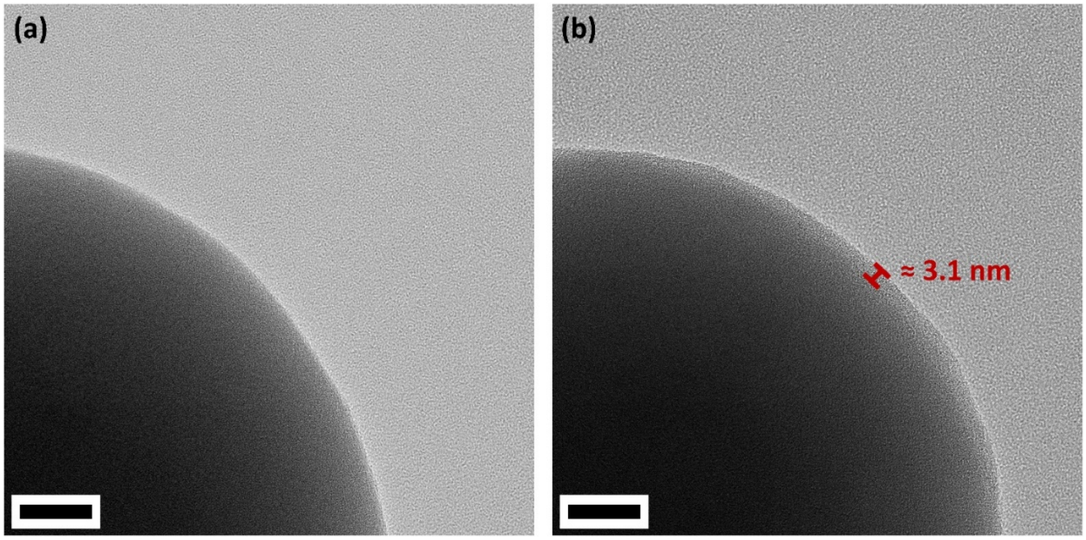

Figure S11. TEM images of (a) **SiO_2** and (b) **MIPTBA@SiO_2** to demonstrate the MIP shell on the particle surface. Scale bar = 20 nm.

13 Kinetics of binding for MIPTBA@SiO_2 and MIPTHA@SiO_2

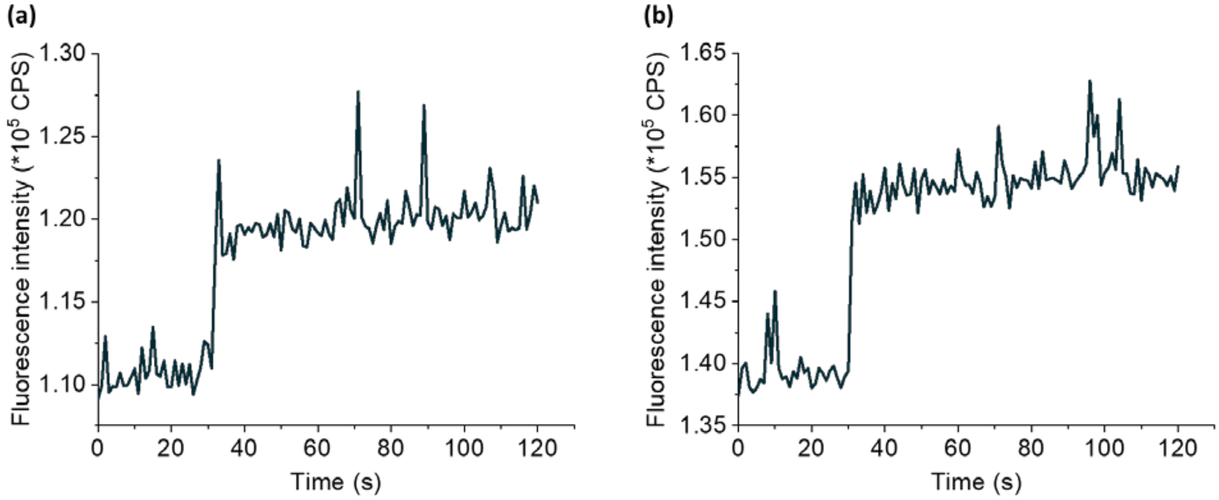

Figure S12. Kinetics of interaction of 2 mL of 1 mg mL^{-1} (a) **MIPTBA@SiO_2** and (b) **MIPTHA@SiO_2** in chloroform after addition of 24 µM GPS-TBA and GPS-THA respectively to the particle suspensions in chloroform. The suspensions were mixed for 30 seconds for equilibration, after which the template solution was added and mixing continued. Similar results were expected for the dNIP particles due to comparable shell thickness. The signal increase upon addition of template was virtually instantaneous in both cases. Kinetics recorded at λ_{ex} = 385 nm and λ_{em} = 491 nm.

14 Determination of amount of I incorporated into MIP and dNIP particles

Following MIP synthesis, a 15–20 nm shift in the wavelength of maximum absorption of **I** in MIP and dNIP particles was observed compared to **I** in dilute solution. Consequently, the molar absorption coefficient of fluorescent crosslinker **I** at a specified wavelength in chloroform could not be used to accurately determine the concentration of **I** in the MIP and dNIP particles. Instead, the area under the longest wavelength band of the absorption spectrum of a known

concentration of **I** in chloroform was used as reference for the determination. Absorption spectra of three stock solutions of **I** in chloroform (7.39 µM) were recorded with two replicates in a quartz cuvette with 1 cm optical path length (Figure S13). Absorption spectra of known particle concentrations of the MIP and dNIP particles were recorded in chloroform in 2 replicates in a quartz cuvette with 1 cm optical path length. For **MIPTBA@SiO₂** and **NIPTBA@SiO₂**, the absorption data at the end point of the titration was used, since prior to titration a proportion of **I⁻** was present, which was converted to **I** after titration.

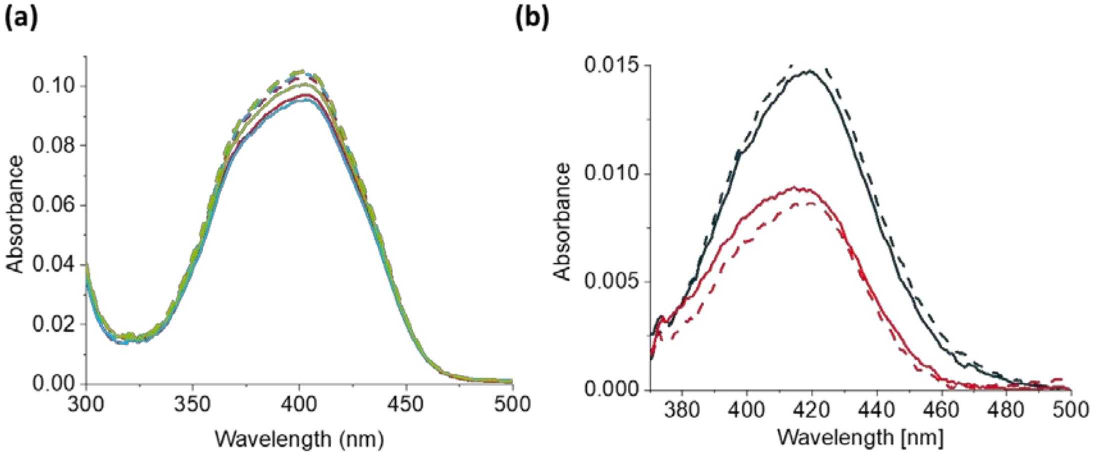

Figure S13. (a) Absorption spectra of three stock solution in two replicates of fluorescent crosslinker **I** (7.39 µM) in chloroform. (b) Corrected absorption spectra of duplicates of 2 mL of 1 mg mL^{-1} of **MIPTBA@SiO$_2$** (black lines) and **NIPTBA@SiO$_2$** (red lines) in chloroform. Spectra for **MIPTHA@SiO$_2$** and **NIPTHA@SiO$_2$** in chloroform were similar.

15 Fluorescence titration spectra of reproduced MIPTBA@SiO$_2$ with GPS-TBA and MIPTHA@SiO$_2$ with GPS-THA

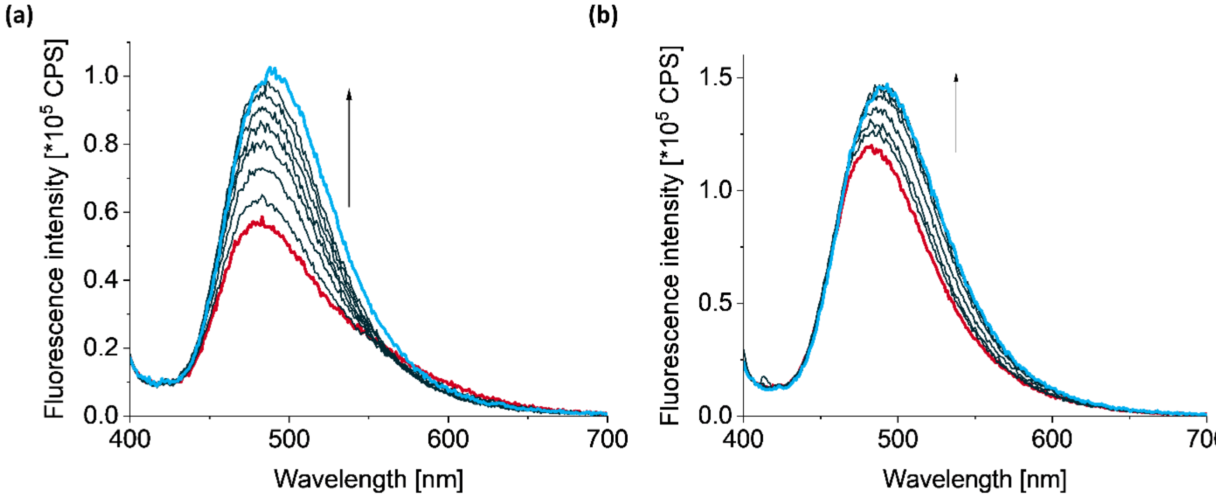

Figure S14. Fluorescence titration spectra of 2 mL each of 1 mg mL^{-1} suspensions of reproduced batches of (a) MIPTBA@SiO$_2$ following addition of up to 87 µM of GPS-TBA and (b) MIPTHA@SiO$_2$ following addition of up to 74 µM of GPS-THA in chloroform. λ_{ex} = 385 nm.

16 Fluorescence titration spectra of MIPTBA@SiO$_2$ with MPPA-TBA

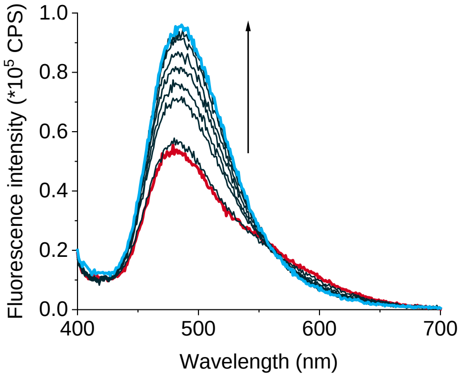

Figure S15. Fluorescence titration spectra of 2 mL of 1 mg mL^{-1} of **MIPTBA@SiO$_2$** following addition of up to 87 µM of MPPA-TBA in chloroform. λ_{ex} = 385 nm.

17 Fluorescence titration spectra of MIPTHA@SiO$_2$ with competitors

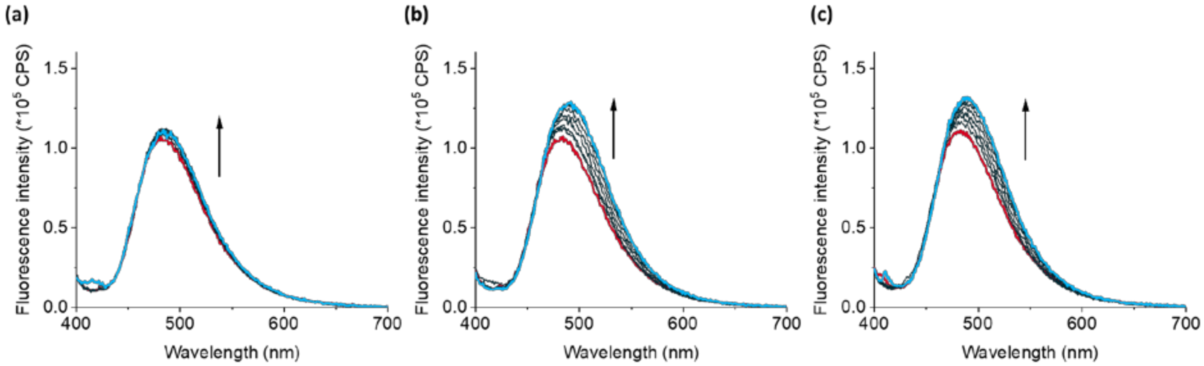

Figure S16. Fluorescence titration spectra of 2 mL of 1 mg mL^{-1} of **MIPTHA@SiO$_2$** each following addition of up to 74 µM of (a) MPPA-THA, (b) 2,4-D-THA and (c) dicamba-THA in chloroform. λ_{ex} = 385 nm.

18 Determination of LOB and LOD of MIPTBA@SiO$_2$ and MIPTHA@SiO$_2$

The parameters Limit of Blank (LOB) and Limit of Detection (LOD) describe the smallest concentration of a sample that can be reliably measured by an analytical procedure [3]. LOB is defined as the highest putative analyte concentration expected to be found when replicates of a blank sample in absence of the analyte are measured. LOD is defined as the lowest analyte concentration likely to be reliably distinguished from the LOB and at which detection is feasible.

The absolute values of the fluorescence emission data at 491 nm following titration with increasing amounts of corresponding template (GPS-TBA or GPS-THA) were fit using a logistic function (Figure S17). From the fitting equation, the concentration corresponding to the fluorescence response of three blank measurements was used to determine the limit of blank (LOB). Three repeat measurements of the lowest concentration used (0.5 µM for GPS-TBA and

2.49 µM for GPS-THA) were used to determine the limit of detection (LOD). LOB and LOD were calculated as below [3].

$\mathbf{LOB} = \bar{\mathbf{x}}^{\mathbf{0}} + \mathbf{1.645SD^{LOB}}$, where $\bar{\mathbf{x}}^{\mathbf{0}}$ is the concentration corresponding to the average response of 3 blank measurements (MIP particles without analyte) and $\mathbf{SD^{LOB}}$ is the standard deviation of these measurements

$\mathbf{LOD} = \mathbf{LOB} + \mathbf{1.645SD^{LOD}}$, where $\mathbf{SD^{LOD}}$ is the standard deviation of 3 measurements of the lowest concentration of analyte used.

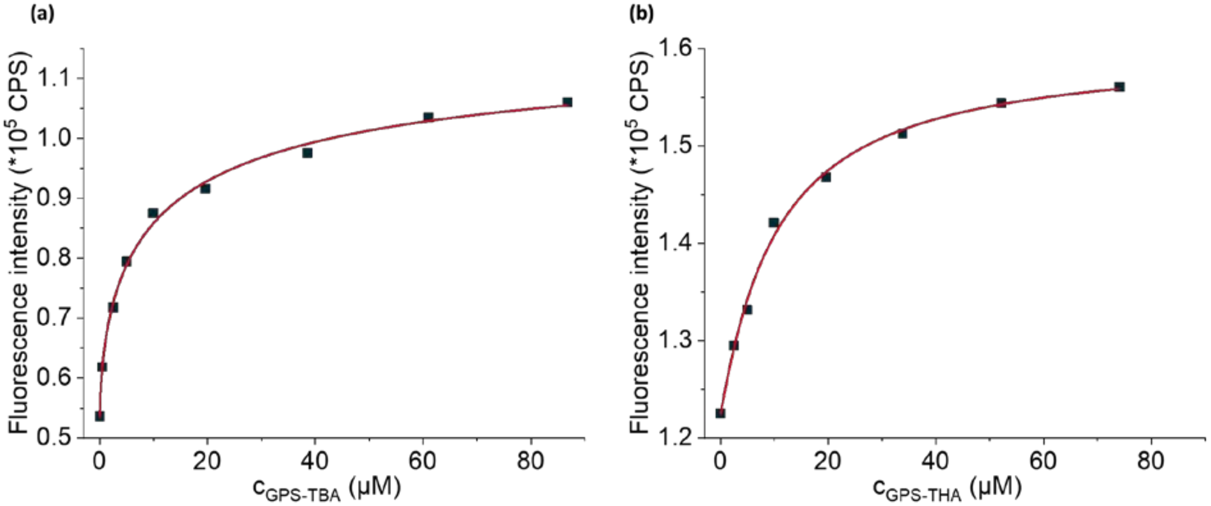

Figure S17. Logistic curve fitting using fluorescence emission intensity at 491 nm (λ_{ex} = 385 nm) for (a) **MIPTBA@SiO₂** and (b) **MIPTHA@SiO₂**. 2 mL of 1 mg mL^{-1} suspensions of particles in chloroform were used.

19 Fluorescence titration spectra of MIPTBA@SiO_2 with GPS-TBA in the biphasic assay

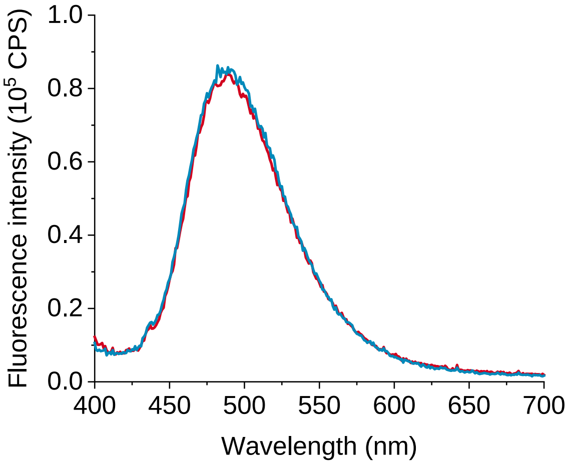

Figure S18. Fluorescence titration spectra of 2 mL of 1 mg mL^{-1} of **MIPTBA@SiO_2** suspended in chloroform (red line) following addition of up to 60 µM of an aqueous solution of GPS-TBA to 1 mL of Milli-Q water on the upper aqueous layer in a biphasic assay. No fluorescence increases were observed. λ_{ex} = 385 nm.

20 Fluorescence titration spectra of MIPTHA@SiO₂ with MPPA-THA in the biphasic assay

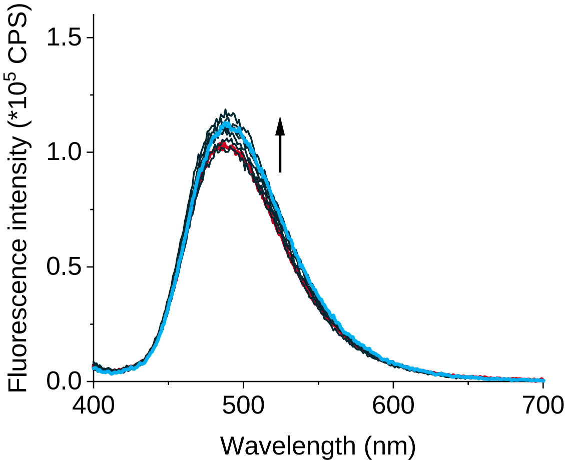

Figure S19. Fluorescence titration spectra of 2 mL of 1 mg mL^{-1} of **MIPTHA@SiO_2** suspended in chloroform (red line) following addition of up to 110 µM of an aqueous solution of MPPA-THA to 1 mL of Milli-Q water on the upper aqueous layer in a biphasic assay. λ_{ex} = 385 nm.

21 Uncertainty budget calculations

Because of the multiplicative and quotient forms of the respective equations and because correlations between the quantities are assumed to be negligible, the summation of the squares of the relative uncertainties was performed [4,5].

For monophasic titrations of MIP and dNIP particles:

i. Weighing of ca. 2.0 mg of particles (balance Mettler Toledo 1 ± 0.01 mg); ${}^{w}_{rel}u$ = 0.5 %
i. Dissolution of particles in 2 mL solvent (Eppendorf Reference pipette ± 0.036 mL); ${}^{d}_{rel}u$ = 1.8 %
ii. Transfer of 2 mL particle suspension to cuvette (Eppendorf Reference pipette ± 0.008 mL); $2\cdot{}^{d2}_{rel}u$ = 2·0.8 %; contribution from cell length (± 0.01 mm); ${}^{L}_{rel}u$ = 0.1 %
iii. Addition of template solution to particle suspension during titrations (Eppendorf Reference pipettes); ${}^{t}_{rel}u$ = 1.3 - 2.3 %
iv. Relative uncertainty of emission; ${}^{e}_{rel}u = 0.6\ \%$
v. Repeat uncertainty of three measurements; ${}^{r}_{rel}u$ = 1.9 – 5.3 %

Total relative error: $\sqrt{({}^{w}_{rel}u^2 + {}^{d}_{rel}u^2 + 2 * {}^{d2}_{rel}u^2 + {}^{L}_{rel}u^2 + {}^{t}_{rel}u^2 + {}^{e}_{rel}u^2 + {}^{r}_{rel}u^2)}$ = 3.2 – 5.7 %

For biphasic titrations of MIP and dNIP particles without repeat uncertainty:

ii. Weighing of ca. 2.0 mg of particles (balance Mettler Toledo 1 ± 0.01 mg); ${}_{\mathrm{rel}}^{\mathrm{w}}\mathrm{u}$ = 0.5 %
vi. Dissolution of particles in 2 mL solvent (Eppendorf Reference pipette ± 0.036 mL); ${}_{\mathrm{rel}}^{\mathrm{d}}\mathrm{u}$ = 1.8 %
vii. Transfer of 2 mL particle suspension to cuvette (Eppendorf Reference pipette ± 0.008 mL); $2\cdot{}_{\mathrm{rel}}^{\mathrm{d2}}\mathrm{u}$ = 2·0.8 %; contribution from cell length (± 0.01 mm); ${}_{\mathrm{rel}}^{\mathrm{L}}\mathrm{u}$ = 0.1 %
viii. Addition of 1 mL water; ${}_{\mathrm{rel}}^{\mathrm{d3}}\mathrm{u}$ = 0.8 %
ix. Addition of template solution to particle suspension during titrations (Eppendorf Reference pipettes); ${}_{\mathrm{rel}}^{\mathrm{t}}\mathrm{u}$ = 1.0 - 2.3 %
x. Relative uncertainty of emission; ${}_{\mathrm{rel}}^{\mathrm{e}}\mathrm{u} = 0.6\ \%$

Total relative error: $\sqrt{({}_{\mathrm{rel}}^{\mathrm{w}}\mathrm{u}^2 + {}_{\mathrm{rel}}^{\mathrm{d}}\mathrm{u}^2 + 2 * {}_{\mathrm{rel}}^{\mathrm{d2}}\mathrm{u}^2 + {}_{\mathrm{rel}}^{\mathrm{d3}}\mathrm{u}^2 + {}_{\mathrm{rel}}^{\mathrm{L}}\mathrm{u}^2 + {}_{\mathrm{rel}}^{\mathrm{t}}\mathrm{u}^2 + {}_{\mathrm{rel}}^{e}\mathrm{u}^2)}$ = 2.6 – 3.3 %

For biphasic titrations of MIP and dNIP particles with repeat uncertainty:

iii. Weighing of ca. 2.0 mg of particles (balance Mettler Toledo 1 ± 0.01 mg); ${}_{\mathrm{rel}}^{\mathrm{w}}\mathrm{u}$ = 0.5 %
xi. Dissolution of particles in 2 mL solvent (Eppendorf Reference pipette ± 0.036 mL); ${}_{\mathrm{rel}}^{\mathrm{d}}\mathrm{u}$ = 1.8 %
xii. Transfer of 2 mL particle suspension to cuvette (Eppendorf Reference pipette ± 0.008 mL); $2\cdot{}_{\mathrm{rel}}^{\mathrm{d2}}\mathrm{u}$ = 2·0.8 %; contribution from cell length (± 0.01 mm); ${}_{\mathrm{rel}}^{\mathrm{L}}\mathrm{u}$ = 0.1 %
xiii. Addition of 1 mL water; ${}_{\mathrm{rel}}^{\mathrm{d3}}\mathrm{u}$ = 0.8 %
xiv. Addition of template solution to particle suspension during titrations (Eppendorf Reference pipettes); ${}_{\mathrm{rel}}^{\mathrm{t}}\mathrm{u}$ = 1.0 - 2.3 %
xv. Relative uncertainty of emission; ${}_{\mathrm{rel}}^{\mathrm{e}}\mathrm{u} = 0.6\ \%$
xvi. Repeat uncertainty of three measurements; ${}_{\mathrm{rel}}^{\mathrm{r}}\mathrm{u}$ = 50 %

Total relative error: $\sqrt{({}_{\mathrm{rel}}^{\mathrm{w}}\mathrm{u}^2 + {}_{\mathrm{rel}}^{\mathrm{d}}\mathrm{u}^2 + 2 * {}_{\mathrm{rel}}^{\mathrm{d2}}\mathrm{u}^2 + {}_{\mathrm{rel}}^{\mathrm{d3}}\mathrm{u}^2 + {}_{\mathrm{rel}}^{\mathrm{L}}\mathrm{u}^2 + {}_{\mathrm{rel}}^{\mathrm{t}}\mathrm{u}^2 + {}_{\mathrm{rel}}^{e}\mathrm{u}^2 + {}_{\mathrm{rel}}^{\mathrm{r}}\mathrm{u}^2)}$ = 50.1 %

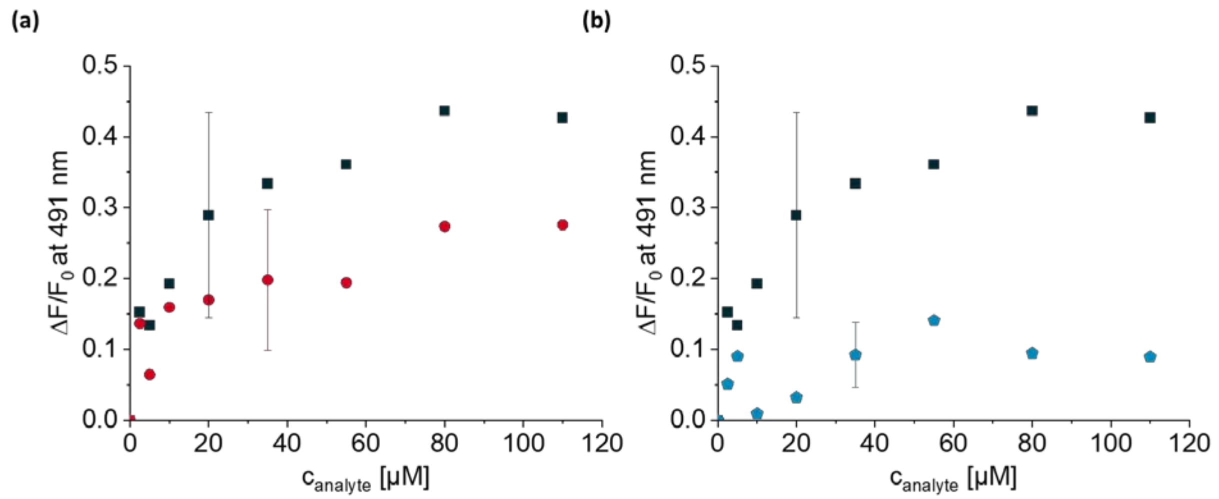

Figure S20. (a) Relative fluorescence changes, $\frac{\Delta F}{F_0}$, at 491 nm of **MIPTHA@SiO$_2$** (black squares) and **dNIPTHA@SiO$_2$** (red circles) upon titration with GPS-THA. (b) Relative fluorescence increases of **MIPTHA@SiO$_2$** upon titration with GPS-THA (black squares) and MPPA-THA (blue pentagons). 2 mL each of 1 mg mL^{-1} suspensions of particles was used in each experiment, with 1 mL of Milli-Q water added above the chloroform phase. The error bars are given with the contribution from the repetition of the experiments. Because Figure S20 shows the results of biphasic experiments carried out in conventional 10x10 mm cuvettes these fluctuations stem from the mixing process and microscopically non-complete phase separation or small droplets still adhering to the cuvette wall through which the fluorescence is measured (90° geometry of the fluorometer) after the solution was allowed to settle and phase-separate. Because the mixing/phase separation procedure was repeated after each step of addition, fluctuations are significant. However, in order to be able to obtain detailed spectral information at every point of template addition, it was necessary to carry out these experiments with a fluorometer. The experiments have been performed several times, revealing that the major contribution to the error is the repeat uncertainty based on the above-mentioned issues. However, as we have recently shown [6], such problems with 10x10 mm cuvettes can be strongly reduced when working with microfluidic setups. λ_{ex} = 385 nm.

22 References

1. Wan, W. *et al.* Ratiometric Fluorescence Detection of Phosphorylated Amino Acids Through Excited-State Proton Transfer by Using Molecularly Imprinted Polymer (MIP) Recognition Nanolayers. *Chem Eur J* **23**, 15974-15983 (2017).

2. Hibbert, D. B. & Thordarson, P. The death of the Job plot, transparency, open science and online tools, uncertainty estimation methods and other developments in supramolecular chemistry data analysis. *Chem Commun* **52**, 12792-12805 (2016).

3. Armbruster, D. A. & Pry, T. Limit of blank, limit of detection and limit of quantitation. *Clin Biochem Rev* **29**, S49-S52 (2008).

4. *Evaluation of measurement data — Guide to the expression of uncertainty in measurement*. (Joint Committee for Guides in Metrology (JCGM), 2008).

5. Rurack, K. & Spieles, M. Fluorescence Quantum Yields of a Series of Red and Near-Infrared Dyes Emitting at 600−1000 nm. *Anal Chem* **83**, 1232-1242 (2011).

6. Wagner, S., Bell, J., Biyikal, M., Gawlitza, K. & Rurack, K. Integrating fluorescent molecularly imprinted polymer (MIP) sensor particles with a modular microfluidic platform for nanomolar small-molecule detection directly in aqueous samples. *Biosens Bioelectron* **99**, 244-250 (2018).
